# InsP_3_R-SEC5 interaction on phagosomes modulates innate immunity to *Candida albicans* by promoting cytosolic Ca^2+^ elevation and TBK1 activity

**DOI:** 10.1186/s12915-018-0507-6

**Published:** 2018-04-27

**Authors:** Long Yang, Wenwen Gu, King-Ho Cheung, Lan Yan, Benjamin Chun-Kit Tong, Yuanying Jiang, Jun Yang

**Affiliations:** 1 School of Pharmacy, Second Military Medical University, 325 Guohe Road, Shanghai, 200433 China; 2 NHFPC Key Laboratory of Reproduction Regulation, Shanghai Institute of Planned Parenthood Research, 2140 Xie Tu Road, Shanghai, 200032 China; 3 School of Chinese Medicine, Hong Kong Baptist University, Hong Kong SAR, China; 4 Jinan Military General Hospital, 25 Shifan Road, Jinan, 250031 China; 5 School of Medicine, Tongji University, Shanghai, 200433 China

**Keywords:** Inositol 1,4,5-trisphosphate receptors (InsP_3_R), Exocyst complex component 2 (SEC5), Tank-binding kinase 1 (TBK1), Antifungal innate immune response

## Abstract

**Background:**

*Candida albicans* (*C. albicans*) invasion triggers antifungal innate immunity, and the elevation of cytoplasmic Ca^2+^ levels via the inositol 1,4,5-trisphosphate receptor (InsP_3_R) plays a critical role in this process. However, the molecular pathways linking the InsP_3_R-mediated increase in Ca^2+^ and immune responses remain elusive.

**Results:**

In the present study, we find that during *C. albicans* phagocytosis in macrophages, exocyst complex component 2 (SEC5) promotes InsP_3_R channel activity by binding to its C-terminal α-helix (H1), increasing cytosolic Ca^2+^ concentrations ([Ca^2+^]_c_). Immunofluorescence reveals enriched InsP_3_R-SEC5 complex formation on phagosomes, while disruption of the InsP_3_R-SEC5 interaction by recombinant H1 peptides attenuates the InsP_3_R-mediated Ca^2+^ elevation, leading to impaired phagocytosis. Furthermore, we show that *C. albicans* infection promotes the recruitment of Tank-binding kinase 1 (TBK1) by the InsP_3_R-SEC5 interacting complex, leading to the activation of TBK1. Subsequently, activated TBK1 phosphorylates interferon regulatory factor 3 (IRF-3) and mediates type I interferon responses, suggesting that the InsP_3_R-SEC5 interaction may regulate antifungal innate immune responses not only by elevating cytoplasmic Ca^2+^ but also by activating the TBK1-IRF-3 pathway.

**Conclusions:**

Our data have revealed an important role of the InsP_3_R-SEC5 interaction in innate immune responses against *C. albicans*.

**Electronic supplementary material:**

The online version of this article (10.1186/s12915-018-0507-6) contains supplementary material, which is available to authorized users.

## Background

*Candida albicans* (*C. albicans*) is the most common type of infectious fungus and is usually considered to be harmless in healthy individuals. However, systemic fungal infection by *C. albicans* may be life-threatening in immunocompromised patients who have undergone surgery, chemotherapy, or organ or bone marrow transplantation [1, 2]. The body defends against systemic *C. albicans* infection by recruiting innate immune cells, especially macrophages and neutrophils. Early recognition of *C. albicans* invasion by immune cells is mediated by opsonic receptors, such as the Fcγ receptor, and dedicated pattern recognition receptors (PRRs), including Toll-like receptors (TLRs) and C-type lectins [3]. PRRs recognize microbe-specific pathogen-associated molecular patterns (PAMPs) and trigger multiple intracellular signaling pathways to orchestrate a pathogen-specific host immune response [3]. Previous studies have suggested that phospholipase Cγ2 (PLCγ2) is a key component in the C-type lectin-mediated immune response against fungal infection [4, 5]. Activation of PLCγ2 cleaves the membrane phospholipid PI(4,5)P_2_ to generate inositol 1,4,5-trisphosphate (InsP_3_), which then triggers the activation of the inositol 1,4,5-trisphosphate receptor (InsP_3_R) signaling pathway [6]. InsP_3_R is a major endoplasmic reticulum (ER) Ca^2+^ channel, and its activation promotes the release of Ca^2+^ from intracellular Ca^2+^ stores, resulting in an elevation of cytosolic Ca^2+^ concentrations ([Ca^2+^]_c_) [7, 8]. Vaeth et al*.* reported that such intracellular Ca^2+^ elevation in macrophages and dendritic cells is required for the activation of key downstream antifungal functions such as phagocytosis, cytokine production, and inflammasome activation [9]. These studies suggest that InsP_3_Rs may play an indispensable role in regulating antifungal immunity. However, the molecular mechanisms linking InsP_3_R to antifungal innate immunity have yet to be determined.

Three homologous isoforms of InsP_3_R are ubiquitously expressed in mammalian tissues, namely InsP_3_R1, InsP_3_R2, and InsP_3_R3 [7]. Their amino acid sequences are 60–80% identical, showing particularly strong homology in the C-terminal tails of the receptors [7]. Despite the fact that the InsP_3_-binding properties, ion permeation, and gating behavior of each isoform are distinct [7], it appears in general that the receptors require Ca^2+^ binding and that the bound Ca^2+^ modulates the temporal and spatial Ca^2+^ release from intracellular Ca^2+^ stores [10]. Each InsP_3_R monomer consists of ~ 2600 amino acids divided into three functional domains: the N-terminal InsP_3_-binding domain, the “regulatory”/“coupling” domain, and the C-terminal channel domain. The C-terminal tail is referred to as the gatekeeper region of the receptor [11–13]; although it is at some distance from the InsP_3_-binding domain, its interactions with several proteins have been shown to affect the sensitivity of the channel to InsP_3_. For example, proteins such as Bcl-2 and Bcl-X_L_ have been shown to interact with the carboxyl terminus of InsP_3_R to regulate InsP_3_R-mediated Ca^2+^ signals that play important regulatory roles in lymphocyte (T cell and B cell) development and selection [14, 15]. To identify novel InsP_3_R-binding partners involved in antifungal innate immunity, we performed yeast two-hybrid screens of a human brain complementary DNA (cDNA) library using a bait corresponding to the cytoplasmic tail of the type 1 InsP_3_R carboxy terminus, identifying exocyst complex component 2 (SEC5) as a protein that binds to the InsP_3_R C-terminus.

SEC5, a member of the hetero-octameric exocyst complex, regulates the targeting and tethering of selective secretory vesicles to specialized dynamic plasma membrane domains [15]. Moreover, SEC5 modulates a number of physiological processes in addition to, but separate from, its role as a component of the exocyst [16]. It has been shown that SEC5 associates with phagosomes involved in the uptake of *Staphylococcus aureus* [17], and it is also involved in Tank-binding kinase 1 (TBK1)-dependent type I interferon innate immune responses against viral infections [18, 19]. Although type I interferons (IFN-α/β) have traditionally been the hallmark of immune response to bacteria and viruses, their importance in host defense against *C. albicans* was recently reported [20–24]. However, the underlying molecular mechanisms are not fully defined.

To this end, we hypothesized that the interaction between SEC5 and InsP_3_R may be involved in the regulation of antifungal innate immunity by activating the InsP_3_R-mediated Ca^2+^ signal and/or the TBK1-dependent immune response. Here, we found that the binding of SEC5 to InsP_3_R promoted its channel activity, increasing cytosolic [Ca^2+^]_c_ in macrophages during the phagocytosis of *C. albicans*. We also showed that the InsP_3_R-SEC5 interaction promoted the recruitment of TBK1 by SEC5, thus forming an InsP_3_R-SEC5-TBK1 complex to modulate *C. albicans*-induced type I interferon innate immune responses.

## Results

### InsP_3_R-modulated Ca^2+^ is required for *C. albicans* phagocytosis in mouse macrophages

**Fig. 1:**
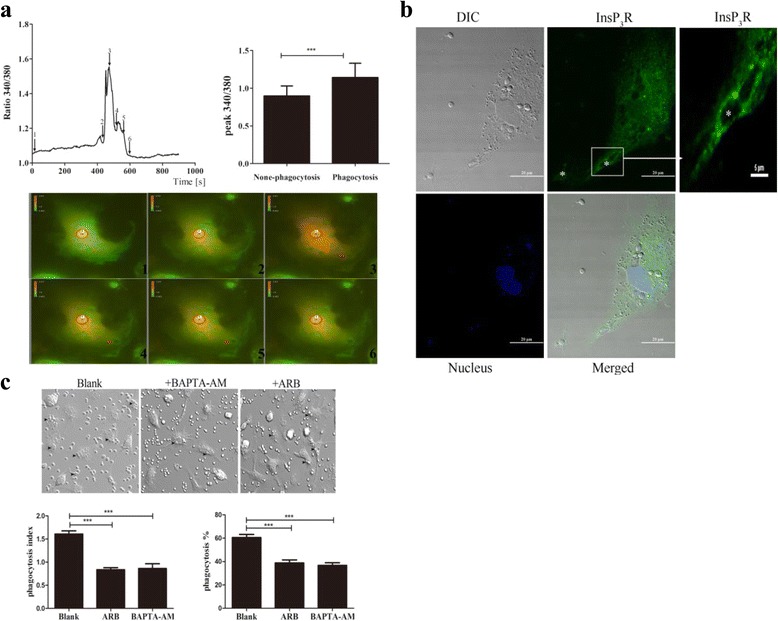
InsP_3_R-modulated Ca^2+^ is required for *C. albicans* phagocytosis. **a** Confocal images depicting the changes of cytosolic Ca^2+^ concentrations ([Ca^2+^]_c_) in BMDMs incubated with *C. albicans.* The *upper line* depicts individual cellular [Ca^2+^]_c_ values. The *upper histogram* depicts the summary of peak [Ca^2+^]_c_ changes during phagocytosis (*n* = 32). Representative snapshots of cytoplasmic Ca^2+^ images were taken at different times during the Ca^2+^ imaging experiments. *Red arrows* indicate internalized *C. albicans*. **b** Confocal images showing the localization of InsP_3_R in BMDMs incubated with *C. albicans. Asterisks* indicate *C. albicans*. **c** Effect of InsP_3_R-modulated Ca^2+^ release on the phagocytosis of BMDM induced by *C. albicans*. Macrophages were co-cultured with *C. albicans* for 30 min. Representative live-cell video microscopy still images showing variation in the number of *C. albicans* engulfed by individual macrophages at 30 min. *Arrows* indicate the BMDMs that took up *C. albicans*. The phagocytosis index was calculated as the average number of *C. albicans* ingested by BMDMs during 30 min. Phagocytosis percentages represent the percentage of BMDMs undergoing *C. albicans* internalization during 30 min. Data are summarized as the mean ± SEM from three experiments (***p < 0.001)


Innate immune cells eliminate pathogens by phagocytosis, a receptor-mediated, actin-dependent process that requires calcium (Ca^2+^) [6, 25]. To evaluate the role of InsP_3_R-modulated Ca^2+^ signals in phagocytosis, we first monitored changes in cytoplasmic [Ca^2+^]_c_ during phagocytosis by bone marrow-derived macrophages (BMDMs). *C. albicans* were added to BMDM, and [Ca^2+^]_c_ levels were recorded in individual macrophages for 10–20 min during the course of phagocytosis. We found that *C. albicans* transiently triggered [Ca^2+^]_c_ elevation, which was followed by the uptake of *C. albicans* (Fig. 1a, Additional file 1: Movie S1). We examined BMDMs internalizing *C. albicans* for 15 min through immunofluorescence microscopy and found that InsP_3_R was localized to phagosomes engulfing *C. albicans* (Fig. 1b). It has been demonstrated that the uptake of large particles is a Ca^2+^-dependent process [26]. To determine if the InsP_3_R-mediated Ca^2+^ signal is involved in phagocytosis, we treated BMDMs with the membrane-permeable Ca^2+^ chelator BAPTA-AM. Pretreatment with BAPTA-AM inhibited *C. albicans* uptake (Fig. 1c). Consistently, inhibition of InsP_3_R activity by preincubation with araguspongin B (ARB, a specific InsP_3_R inhibitor) [27, 28] significantly inhibited *C. albicans*-induced phagocytosis (Fig. 1c). Together, these results suggest that phagocytosis of *C. albicans* triggered Ca^2+^ release through InsP_3_R, and InsP_3_R-modulated Ca^2+^ release is essential for the phagocytosis of *C. albicans* in BMDMs.

Additional file 1:**Movie S1.** Live-cell video microscopy movie showing the elevation of [Ca^2+^]_c_ in BMDM cells during phagocytosis of *C. albicans*. BMDM cells were stained using Fura-2 AM. BMDMs and *C. albicans* were co-cultured for 30 min at 37°C in complete CO_2_-independent medium. The video shows that cytosolic Ca^2+^ levels in BMDM cells were transiently increased during phagocytosis of *C. albicans.* (MP4 2454 kb)


### InsP_3_R interacts with SEC5 in native cells

**Fig. 2:**
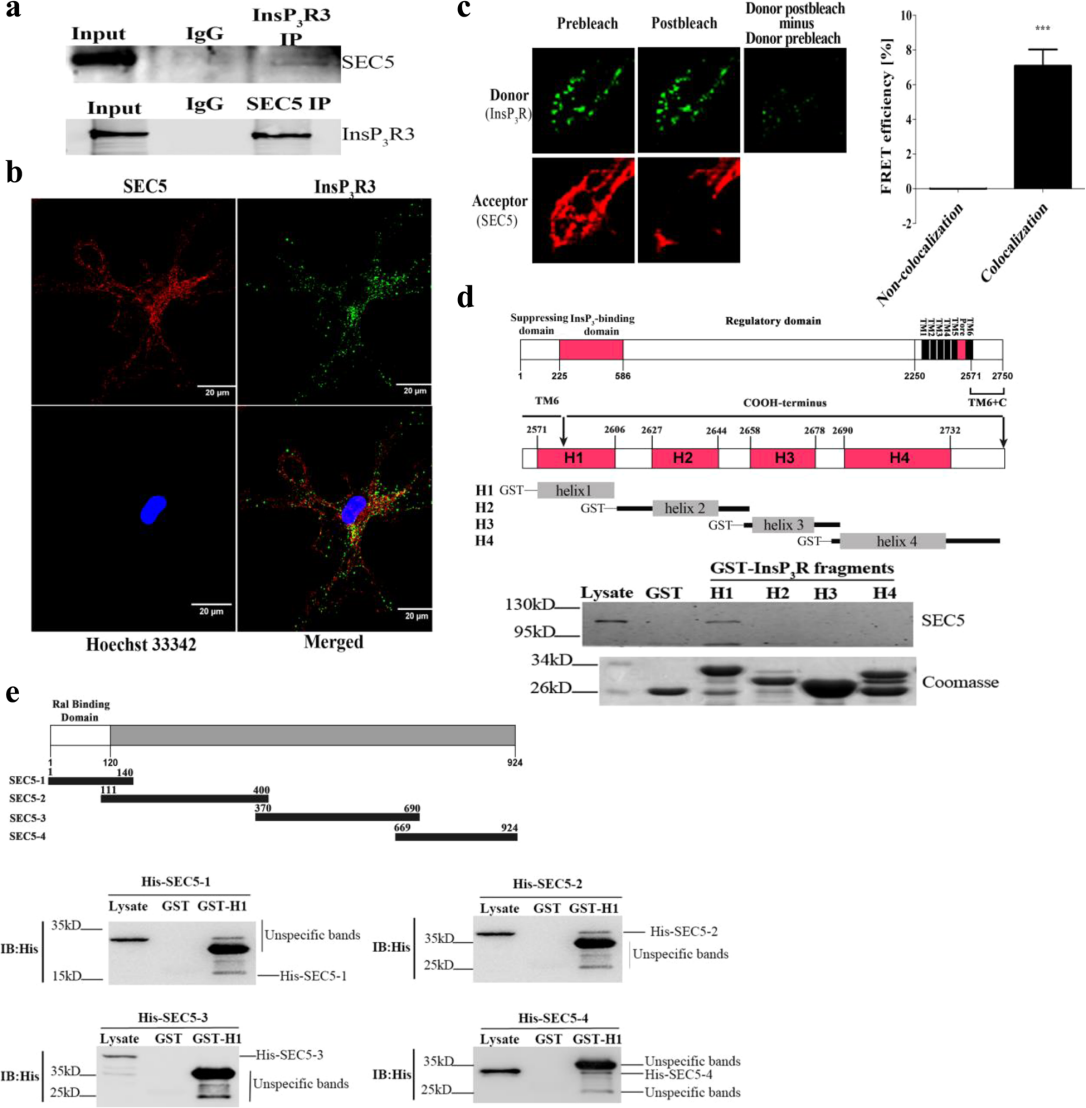
SEC5 interacts with InsP_3_R. **a** Interaction between SEC5 and InsP_3_R3 in RAW264.7 cell lysates. The cell lysates were immunoprecipitated (IP) with control rabbit IgG, anti-SEC5, or anti-InsP_3_R3 antibody. The lysate (*Input*) and IP samples were analyzed by western blotting with the indicated antibodies. **b** Confocal images showing SEC5 and InsP_3_R co**-**localization in BMDM cells (Pearson coefficient = 0.71). A representative resting BMDM cell was immunostained with anti-SEC5 (*red*) and anti-InsP_3_R3 antibodies (*green*). **c** Representative images of FRET donor (Alexa Fluor 488) and acceptor (Cy3). FRET pair intensities before and after Cy3 was photobleached are shown (*left* and *middle columns*). The histogram summarizes FRET efficiency in multiple regions of interest (data are summarized as the mean ± SEM; *n* = 16 for each pair of samples; ***p < 0.001). **d** Schematic illustration of the functional domains of rat InsP_3_R type 1 and GST fusion proteins (H1 to H4) of the InsP_3_R C-terminus (aa 2573–2749) used in SEC5 pull-down assays. A representative western blot depicting the GST pull**-**downs of SEC5 from RAW267.4 cell extracts is shown. The Coomassie blue**-**stained gel shows input GST-tagged H1 to H4 fragments. **e** Schematic diagram of recombinant SEC5 fragments used for testing interactions with InsP_3_R H1 helices (*upper panel*). Representative in vitro pull**-**down assays depict the interaction of different His-tagged SEC5 fragments with GST-InsP_3_R-H1

InsP_3_R is a huge protein whose channel activity can be regulated by numerous interacting proteins [7, 8]. To determine if phagocytosis is regulated by protein interaction with InsP_3_R, we performed yeast two-hybrid screening with a human brain cDNA library. Using the type 1 InsP_3_R carboxy terminus as the bait, we identified the exocyst complex component SEC5 as an InsP_3_R-interacting protein (Additional file 2: Figure S1). Because SEC5 had previously been reported to associate with phagosomes [17], we speculated that the interaction between SEC5 and InsP_3_R may be involved in BMDM phagocytosis against *C. albicans* infection. We then validated the interaction between SEC5 and InsP_3_R in macrophages using co-immunoprecipitation. As shown in Fig. 2a, endogenous SEC5 was successfully co-precipitated from murine macrophage RAW264.7 cell lysates using antibodies against InsP_3_R3. Conversely, InsP_3_R3 was found in the immunoprecipitates with the SEC5 antibodies. Using immunofluorescence staining, we detected that endogenous SEC5 and InsP_3_R were partially co-localized in the cytoplasm of fixed resting BMDM cells (Fig. 2b).

To demonstrate their physical interaction in native BMDM cells, an acceptor photobleaching fluorescence resonance energy transfer (FRET) approach was adopted [29, 30], which measures the dequenching of donor (fluorescein isothiocyanate (FITC)) emission by acceptor (Cy3) photobleaching. After photobleaching with light of the excitation wavelength for the acceptor, the fluorescence intensity in the donor channel increased in co-localized SEC5-InsP_3_R regions, but not in non-co-localized control pairs (Fig. 2c), indicating that SEC5 and InsP_3_R are within 10 nm of each other in those interacting regions. These results provide evidence that SEC5 interacts with InsP_3_R in intact cells.

The C-terminus of InsP_3_R contains four α-helices, denoted H1 to H4, which are of higher homology in all three InsP_3_R isoforms and have been shown to interact with Bcl-2 family proteins [14, 15, 31]. To verify that the InsP_3_R C-terminal helices interact with SEC5, we constructed glutathione *S*-transferase (GST)-tagged type 1 InsP_3_R C-terminal fragments and tested if they could pull down SEC5 from crude cellular extracts. These in vitro pull-down experiments showed that SEC5 was successfully pulled down by GST-H1 only from RAW264.7 cell extracts (Fig. 2d)*.* To delineate the sequences in SEC5 that interact with GST-H1, we divided the full-length SEC5 protein into four His-tagged fragments (SEC5-1 to SEC5-4 in Fig. 2e). Recombinant GST-H1 peptides pulled down His-tagged SEC5-2 and SEC5-4 fragments, as detected with anti-His antibodies (Fig. 2e). This finding was further confirmed by in vitro direct protein interaction assays (Additional file 3: Figure S2). Our data demonstrated that SEC5 interacted with the C-terminus of InsP_3_R in native cells, and revealed their respective interacting sequences.

### SEC5 modulates InsP_3_R channel gating

**Fig. 3:**
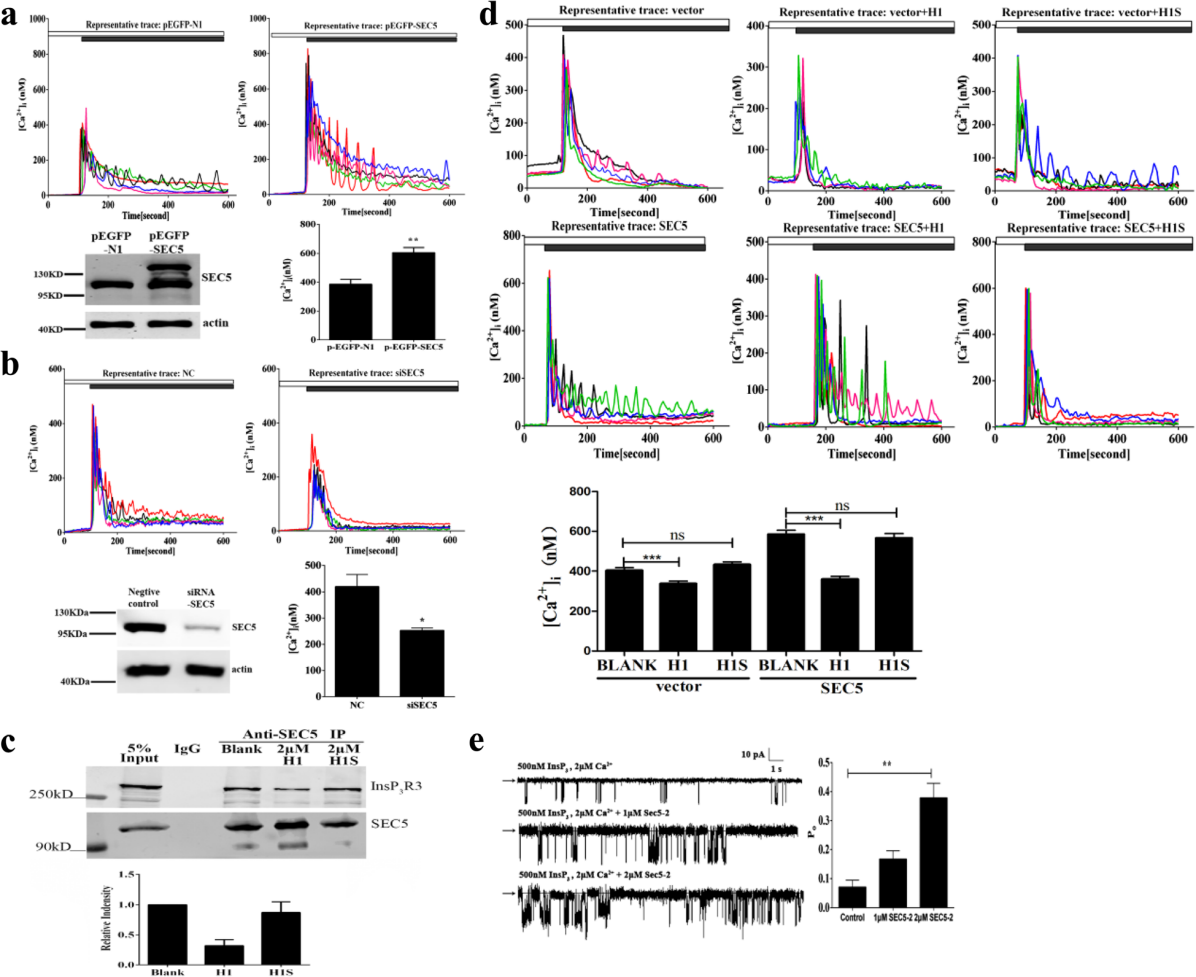
Effects of SEC5 on InsP_3_R channel activity. **a**, **b** Representative Ca^2+^ transients in HEK293 cells stimulated by Ca^2+^-free Hank’s balanced salt solution (*white bar*), followed by addition of 50 μM carbachol to the extracellular solution (*black bar*). **a** Cells were transfected with pEGFP-SEC5 or pEGFP-N1 vectors. Representative western blot depicting the expression of SEC5 in cells transfected with pEGFP-SEC5 or pEGFP**-**N1 vectors. Actin was used as a loading control. **b** Cells were transfected with scramble siRNA or SEC5**-**siRNA. A western blot depicting the knock down of SEC5 by siRNA is shown. Data are summarized as the mean ± SEM from three experiments with more than 100 GFP**-**positive cells analyzed. **c** Co-immunoprecipitation assays in RAW264.7 lysates demonstrated that recombinant H1 peptide competed with endogenous InsP_3_R3 for binding to SEC5. The bar chart summarizes the amount of InsP_3_R3 co-immunoprecipitated by anti-SEC5 antibodies. Data represent the mean ± SEM intensities of the co-immunoprecipitated InsP_3_R3 band from three experiments. **d** Representative Ca^2+^ traces depicting carbachol-induced ER Ca^2+^ release (*black bar*) under the influence of recombinant H1 peptide or H1 scramble peptide. Data are summarized as the mean ± SEM from three experiments with at least 100 cells analyzed. **e** Representative single-channel current traces activated by 500 nM InsP_3_ and 2 μM free Ca^2+^ in the pipette solution. *Arrow* indicates zero**-**current level, whereas downward deflection represents channel openings at pipette voltage = 40 mV. The channel open probability was enhanced by the addition of SEC52 peptide in the pipette solution. Data represent the mean ± SEM for at least three individual nuclear patches. (*p < 0.05, **p < 0.005, ***p < 0.001)

Because Bcl-2 and Bcl-X_L_ have been shown to modulate InsP_3_R channel activity by binding to its C-terminal α-helices [14, 15, 32], we speculated that SEC5 might regulate InsP_3_R channel gating by binding to the H1 helix. To test this, we performed single-cell Ca^2+^ imaging with human embryonic kidney (HEK) 293 cells transfected with an enhanced green fluorescent protein (EGFP)-tagged SEC5 plasmid. Cells transfected with EGFP plasmids were used as a control. Twenty-four hours after transfection, HEK293 cells were loaded with Fura-2 AM, and then [Ca^2+^]_c_ was recorded using a computer-controlled imaging system. We stimulated the cells with carbachol, an acetylcholine analog, to trigger InsP_3_R-mediated Ca^2+^ release [33, 34]. The SEC5-transfected HEK293 cells showed enhanced carbachol-induced Ca^2+^ release compared to that of cells expressing EGFP (Fig. 3a). In contrast, when endogenous SEC5 was knocked down by SEC5-specific small interfering RNA (siRNA) (siRNA-SEC5 in Fig. 3b), the carbachol-induced elevation of [Ca^2+^]_c_ was significantly decreased compared with that of cells transfected with scramble siRNA (Fig. 3b). To confirm that carbachol induces Ca^2+^ release via InsP_3_R, we pretreated cells with the InsP_3_R inhibitor ARB. Carbachol-induced Ca^2+^ release was blocked by ARB when compared with that of untreated control cells (Additional file 4: Figure S3). These results suggested that SEC5 enhances the InsP_3_R-mediated elevation of [Ca^2+^]_c_.

The H1 sequences showed higher homology (more than 90%) in all three InsP_3_R isoforms of different species (Additional file 5: Figure S4). To verify that the effect of SEC5 on InsP_3_R is due to their direct binding, we designed a cell-permeable recombinant peptide, H1-TAT, composed of the HIV-1 Tat protein transduction domain fused with 25 amino acids derived from the H1 motif of three different InsP_3_R subtypes (Additional file 5: Figure S4). A scrambled H1-TAT peptide (H1S-TAT) was synthesized and used as a control (Additional file 5: Figure S4). The effect of the peptides on the SEC5-InsP_3_R interaction was tested in a competitive co-immunoprecipitation assay by monitoring the formation of the endogenous SEC5-InsP_3_R complex using the SEC5 antibodies. We found that in the presence of H1-TAT, the formation of the endogenous SEC5-InsP_3_R complex was inhibited (Fig. 3c). Furthermore, H1-TAT treatment significantly attenuated the carbachol-induced Ca^2+^ release in SEC5- and EGFP-overexpressing HEK293 cells compared to the control or cells treated with the H1S-TAT scramble peptide (Fig. 3d). These results indicated that the SEC5*-*InsP_3_R interaction plays a role in increasing the agonist-induced Ca^2+^ release from the ER.

To provide direct evidence that SEC5-2 enhances InsP_3_R channel activity, nuclear membrane electrophysiology was performed in a chicken B-lymphoid cell line DT40 expressing recombinant homotetrameric rat type 3 InsP_3_R, in which the endogenous InsP_3_Rs have been genetically knocked out (DT40KO-rInsP_3_R3) [35–37]. With 500 nM InsP_3_ and 2 μM Ca^2+^ in the pipette solution, the InsP_3_R channel open probability (*P*_o_) was 0.07±0.024. Using the same ligand-stimulating conditions and in the presence of purified SEC5-2 recombinant peptides, the channel *P*_o_ was significantly enhanced in a dose-dependent manner (Fig. 3e; 1 μM, 0.17±0.029 and 2 μM, 0.38±0.051). Taken together, these results demonstrated that SEC5 modulates InsP_3_R Ca^2+^ channel gating through direct interaction.

### The SEC5-InsP_3_R interaction modulates macrophage phagocytosis

**Fig. 4:**
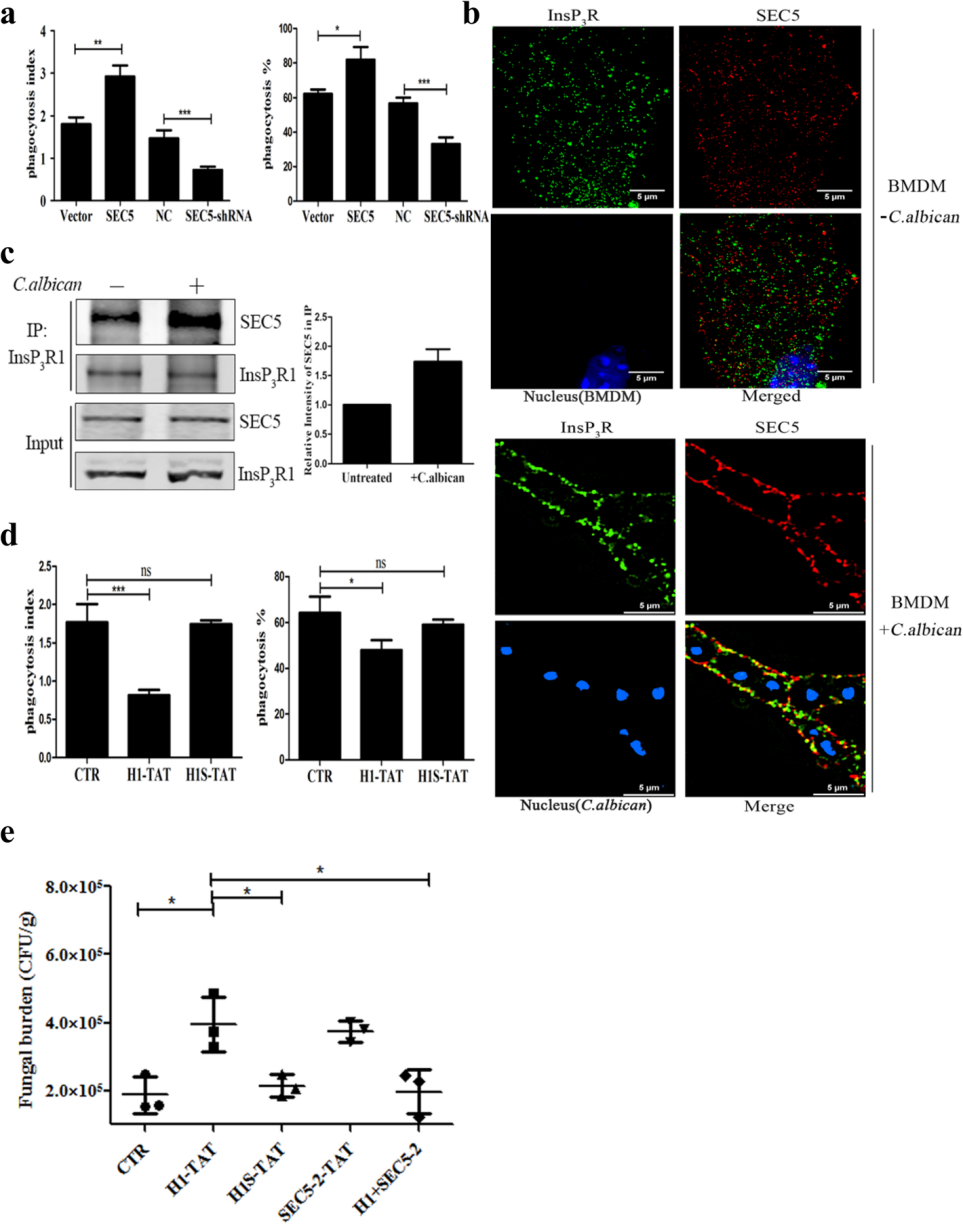
SEC5-InsP_3_R interaction facilitates macrophage phagocytosis. **a** Effect of SEC5 expression on the phagocytosis of *C. albicans* by BMDMs. Cells were transfected with vectors encoding SEC5**-**EGFP, EGFP, SEC5**-**siRNA, or scramble siRNA, and cells were incubated with *C. albicans* for 30 min (MOI = 10). Phagocytosis index was calculated as the average number of *C. albicans* ingested by BMDM cells during 30 min. Phagocytosis percentage represents the percentage of BMDMs undergoing *C. albicans* internalization during 30 min. Data are summarized as the mean ± SEM from three experiments. **b** Confocal images depicting the localization of SEC5 and InsP_3_R in resting (*upper panel*) or activated (*C. albicans-*infected; *lower panel*) BMDM cells. **c** Enhanced co-immunoprecipitation of endogenous SEC5 using antibodies against InsP_3_R1 in RAW264.7 cell lysates treated with *C. albicans* (MOI = 10) for 1 h. Lysates from cells not infected with *C. albicans* were used as a negative control. Quantification of SEC5 immunoreactive bands, normalized to their respective InsP_3_R1 bands, is shown in the *right panel*. Bars represent fold change in SEC5 intensity (mean ± SEM from three experiments). **d** Effect of recombinant cell-permeable H1-TAT peptide on BMDM phagocytosis. BMDM cells were pretreated with H1-TAT or scramble H1S-TAT (2 μM) for 6 h using the same protocol as described in **a**. **e** Infected mouse kidney CFU assay. Groups of C57B/L6 female mice were injected via lateral tail vein with 1 × 10^5^ CFU of *C. albicans* (SC5314) and treated with the indicated peptides. Kidney CFU assays (*n* = 3 × 3) were performed 24 h after infection. In all panels in **a** and **d**, unless otherwise noted, data represent the mean ± SEM from at least three independent experiments (*p < 0.05, **p < 0.005, ***p < 0.001)


SEC5 has been reported to associate with phagosomes [17], and we speculated that the interaction between SEC5 and InsP_3_R may regulate BMDM phagocytosis against *C. albicans* infection. Thus, we used live-cell video microscopy to record BMDM behavior in real time [38]. Enhancing SEC5 expression in BMDM cells increased the phagocytosis index (average *C. albicans* uptake per BMDM cell) and the ratio of active BMDMs (Fig. 4a). In contrast, knocking down SEC5 expression in BMDMs significantly decreased their phagocytosis activity (Fig. 4a). These results indicated that SEC5 regulates the phagocytosis activity of BMDM cells. In addition, super-resolution structured illumination microscopy (SIM) images indicated that the co-localization of SEC5 and InsP_3_R was enriched in phagosomes when compared with that of resting BMDM and RAW264.7 cells (Fig. 4b, Additional file 6: Figure S5, and Additional file 7: Movie S2). Using type 1 InsP_3_R antibodies to precipitate SEC5 from RAW264.7 cells, we found that *C. albicans* stimulation increased the quantity of endogenous SEC5-InsP_3_R complexes (Fig. 4c), indicating that *C. albicans* stimulation promoted the association of SEC5 and InsP_3_R. In addition, we found that disrupting the SEC5-InsP_3_R interaction with H1-TAT peptides significantly inhibited BMDM phagocytosis when compared with that of cells treated with H1S-TAT (Fig. 4d), suggesting that the interaction between SEC5 and InsP_3_R contributes significantly to the phagocytosis of *C. albicans* in BMDM cells.

Additional file 7:**Movie S2.** Z stack of confocal images depicting the relocalization of SEC5 and InsP_3_R in activated BMDM cells. Related to Fig. 4b. (AVI 1355 kb)


To validate our in vitro findings that the SEC5-InsP_3_R interaction is involved in antifungal innate immunity, we examined the effects of the H1-TAT peptide in a murine model of systemic candidiasis. C57BL/6 mice were injected via the lateral tail vein with 1 × 10^5^ colony-forming units (CFUs) of *C. albicans*, followed by intraperitoneal (i.p.) injection with the indicated cell-permeable peptides. Compared to the controls, the kidney fungal burdens were significantly higher in the groups treated with H1-TAT or SEC5-2 peptides, while co-injection of SEC5-2 and H1-TAT neutralized their respective toxic effects (Fig. 4e). These data indicated that the SEC5-InsP_3_R interaction promotes innate immunity against *C. albicans* infection in vivo, while disturbing their interaction weakens innate immunity against *C. albicans*.

### SEC5 and TBK1 regulate IFN-β production in response to *C. albicans*

**Fig. 5:**
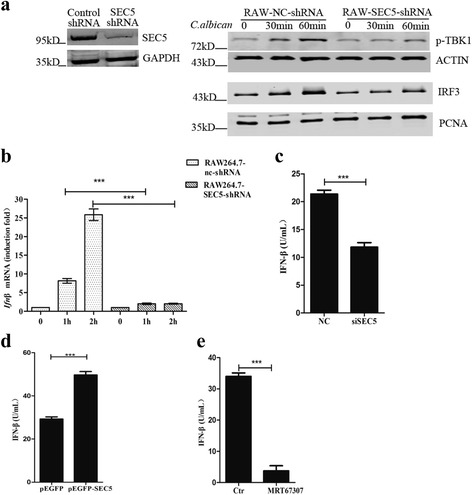
SEC5 and TBK1 modulate *C. albicans-*induced IFN-β production. **a** Representative immunoblots showing the amount of phosphorylated TBK1 (*p*-*TBK1*) in the cytosolic fraction and IRF-3 in the nuclear fraction from RAW264.7 cells at different time points of *C. albicans* stimulation (MOI = 10). Cells were pretreated with SEC5-shRNA or scramble shRNA (*NC*-*shRNA*), and western blots for SEC5 after shRNA treatments are shown for comparison. **b** Expression of IFN-β in *C. albicans*-infected RAW264.7 cells pretreated with SEC5-shRNA or scramble shRNA was measured by quantitative real-time PCR at the indicated time points. Data were normalized to the expression of β-actin. **c** BMDMs were transfected with SEC5-EGFP or EGFP vectors for 48 h, stimulated with *C. albicans* for 4 h, and then IFN-β production was analyzed by ELISA. **d** BMDMs were treated with SEC5-siRNA or scramble siRNA for 48 h, stimulated with *C. albicans* for 4 h, and then IFN**-**β production was analyzed by ELISA. **e** BMDMs were treated with 20 nM MRT67307 for 2 h, stimulated with *C. albicans* for 4 h, and then IFN**-**β production was analyzed by ELISA. Data are expressed as the mean ± SEM from at least three independent experiments (***p < 0.001)

Type I interferon (IFNα/β) is crucial for responding to infections through its antiviral properties and by its function in coordinating the immunocompetent cells involved in antiviral or antibacterial immunity [20]. Recent studies have shown that IFN-β produced in response to *C. albicans* infection is crucial for defending against candidiasis [21–24]. SEC5 has been shown to modulate the TBK1-IRF-3 signaling-dependent type I interferon responses against viral infections [18, 19]. To determine if the SEC5-TBK1-IRF-3 signaling cascade is involved in *C. albicans* infection, we analyzed the phosphorylation of TBK1 (p-TBK1) and IRF-3 nuclear translocation during fungal infection, the results of which showed that *C. albicans* stimulation increased p-TBK1 levels in RAW264.7 cells (Fig. 5a). It has been shown that activated TBK1 phosphorylates IRF-3 and promotes its nuclear translocation [17]. In agreement with this, we found enhanced IRF-3 levels in the nuclear fraction of RAW264.7 lysates (Fig. 5a). Conversely, when SEC5 expression was knocked down by small hairpin RNA (shRNA), TBK1 phosphorylation and the nuclear fraction of IRF-3 remained unchanged during *C. albicans* stimulation (Fig. 5a). We also assessed the production of IFN-β upon *C. albicans* stimulation by quantitative real-time polymerase chain reaction (PCR) and enzyme-linked immunosorbent assay (ELISA) (Fig. 5b and c). The production of IFN-β transcripts and cytokines was increased upon fungal infection, while knocking down SEC5 inhibited the production of INF-β (Fig. 5b and c). Furthermore, overexpression of SEC5 in RAW264.7 significantly enhanced the production of INF-β (Fig. 5d), while treating RAW264.7 cells with the TBK1 inhibitor MRT67307 markedly inhibited the production of IFN- β (Fig. 5e). Altogether, these data demonstrated that *C. albicans* infection activates TBK1, and the subsequent nuclear translocation of IRF-3 promotes INF-β production in a SEC5-dependent manner.

### InsP_3_R regulates SEC5-TBK1-IRF-3 signaling

**Fig. 6:**
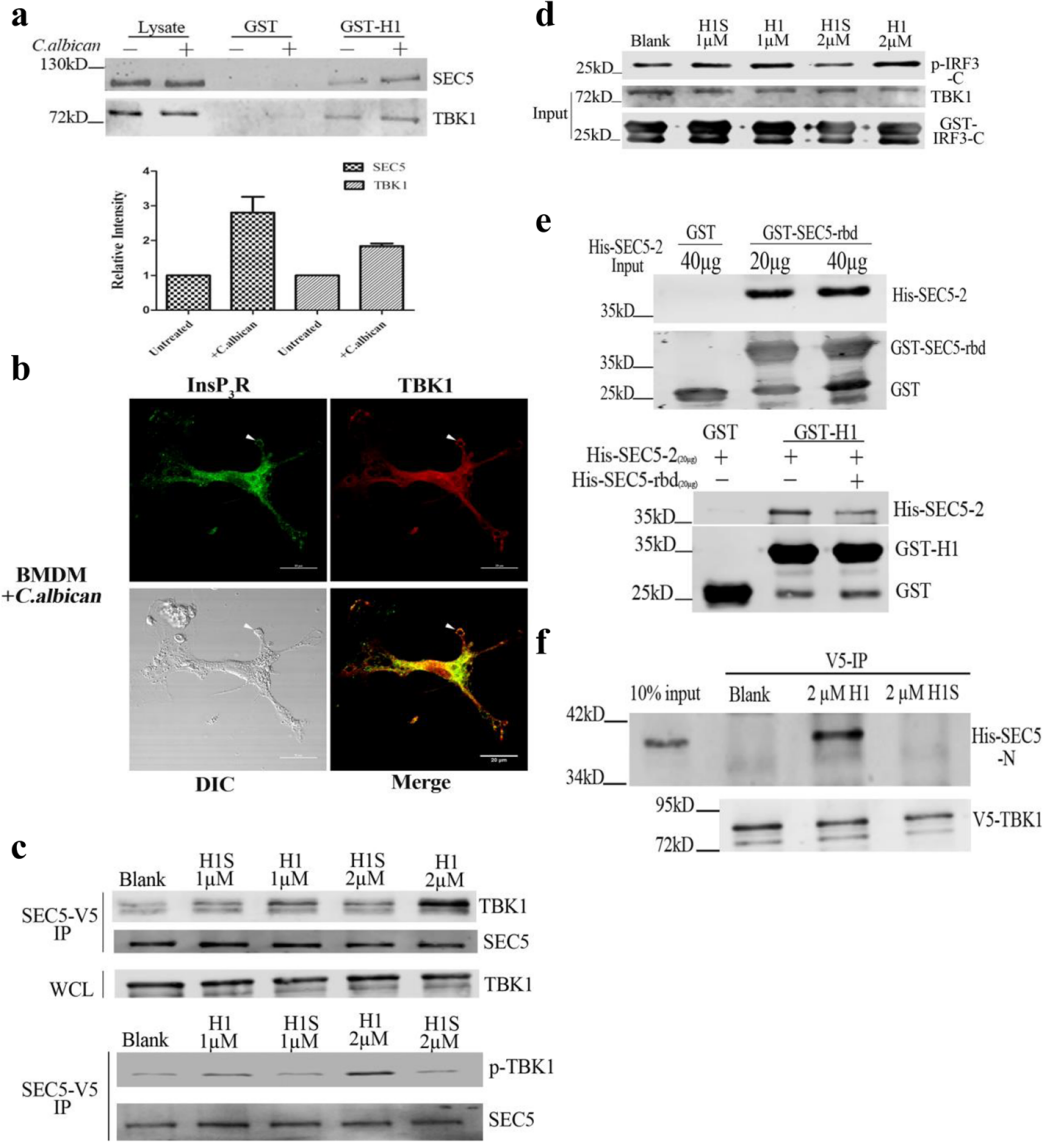
SEC5-InsP_3_R interaction influences TBK1-IRF-3 signaling cascade. **a** In vitro pull-down of SEC5 and TBK1 in *C. albicans*-stimulated (MOI = 10) RAW264.7 cell lysates by GST-H1 or GST. Quantification of SEC5 and TBK1 immunoblot signal intensities from GST-H1 pull-down experiments is shown. Data are summarized as the mean ± SEM from three separated experiments. **b** Confocal images depicting the co-localization of TBK1 and InsP_3_R on phagosomes (Pearson coefficient = 0.87). *White arrow* indicates phagosome. **c** Immunoblots showing TBK1 and p-TBK1 immunoprecipitated using V5 antibodies in the presence of H1-TAT or H1S-TAT recombinant peptides. **d** Immunoblots depicting the phosphorylation of recombinant IRF-3-C in the presence of H1 peptides. **e** Immunoblots of in vitro pull-down assays using GST-SEC5-rbd and purified His-SEC5-2. Purified SEC5-rbd inhibited the interaction of SEC5-2 peptides with InsP_3_R-H1. **f** Immunoprecipitation examining the interaction of V5-BTK1 and purified His-SEC5-N in the presence of H1-TAT or H1S-TAT recombinant peptides

As *C. albicans* infection promoted the SEC5-InsP_3_R interaction (Fig. 4c), we examined the consequences of InsP_3_R regulation on the SEC5-TBK1 complex and TBK1 activity. We performed GST pull-down assays using a recombinant InsP_3_R1 H1 helix as the bait. The results indicated that *C. albicans* infection increased the amount of SEC5 and TBK1 pulled down by GST-H1 from RAW264.7 cell lysate (Fig. 6a). Furthermore, we examined BMDMs internalizing *C. albicans* for 15 min by immunofluorescence microscopy and found that InsP_3_R and TBK1 co-localized on phagosomes (Fig. 6b and Additional file 8: Figure S6). These results suggest that *C. albicans* infection promotes the SEC5-InsP_3_R-TBK1 interaction, which may subsequently activate the TBK1-IRF-3 signaling cascade.

We performed co-immunoprecipitation assays to further validate that the SEC5-InsP_3_R interaction is critical for SEC5-InsP_3_R-TBK1 complex formation. In COS-7 cells expressing V5-tagged SEC5, addition of recombinant H1 peptides dose-dependently enhanced the amount of co-immunoprecipitated TBK1 and p-TBK1, whereas H1 scramble peptides did not (Fig. 6c). Intriguingly, InsP_3_R-H1-enhanced formation of the TBK1-SEC5 complex correlated with enhanced TBK1 activity, as measured by in vitro phosphorylation of recombinant IRF-3-C, a direct TBK1 substrate [39, 40] (Fig. 6d). TBK1 was previously reported to interact directly with the Ral-binding domain (rbd) of SEC5 (SEC5-rbd, amino acids (aa) 1–120), which has been shown to bind to SEC5 [18], while the region of SEC5 responsible for the SEC-rbd interaction was unknown. Using GST-SEC5-rbd and purified His-SEC5-2 to perform in vitro pull-down assays, we found that SEC5-rbd bound to SEC5-2, and their interaction prevented InsP_3_R-H1 from binding to SEC5-2 (Fig. 6e). We then hypothesized that InsP_3_R-H1 competes with SEC5-rbd to bind to SEC5-2, which releases SEC5-rbd and allows it to bind to TBK1, thereby increasing TBK1 enzyme activity to subsequently activate the transcription factor IRF-3. To test this hypothesis, we purified the N-terminus of SEC5 (aa 1–440, containing the rbd and the InsP_3_R-binding domain) and V5-TBK1. Immunoprecipitation assays showed that peptide H1 promoted the binding of TBK1 to SEC5-N, unlike the H1 scramble peptide. These data indicate that InsP_3_R regulates the interaction of SEC5 and TBK1. However, neither the binding of SEC5 with InsP_3_R-H1 nor the enzyme activity of TBK1 was Ca^2+^ dependent (Additional file 9: Figure S7).

## Discussion

Invasive candidiasis is the most common cause of systemic fungal infection in hospitalized immunocompromised patients [1, 2]. *C. albicans* infections are rare in healthy individuals, as the infection triggers human host immune mechanisms when it interacts with macrophages, neutrophils, or dendritic cells that subsequently eliminate the invading fungus by phagocytosis [3]. Although the underlying mechanisms for phagocytosis are elusive, it is believed that the kinetics and efficiency of the formation and maturation of phagosomes are promoted by both localized and global cytoplasmic Ca^2+^ elevations that drive the remodeling of F-actins [25, 41, 42]. It has been shown that *C. albicans* infection promotes the activation of PLCγ, which transiently mobilizes Ca^2+^ from the endoplasmic reticulum (ER) and subsequently opens the store-operated Ca^2+^ entry (SOCE) channel to provide a sustained Ca^2+^ signal for initiating host innate immunity [43, 44]. Intriguingly, Vaeth et al. demonstrated that deletion of SOCE did not significantly affect the innate immunity of macrophages and dendritic cells [9]. We found that InsP_3_R localized on phagosomes and mediated Ca^2+^ release from the ER, and these processes were necessary for macrophage phagocytosis (Fig. 1c). In addition to functioning as a Ca^2+^ storage organelle, it has been reported that the ER also provides membranes to phagosomes for the internalization of pathogens by macrophages during phagocytosis [45, 46]. Although the fusion of ER membranes to phagosomes is controversial [47], ER has been observed in close proximity to the base of the phagocytic cup by electron microscopy [45, 48], indicating that the ER or the associated InsP_3_R plays an indispensable role in fungus-induced phagocytosis.

Here, we have proposed a unique molecular mechanism that regulates the host innate immune response against *C. albicans* fungal infections. We showed that the N-terminal region of SEC5 interacts with the α-helix of the InsP_3_R C-terminus (H1) and their interaction regulates both ER Ca^2+^ release and type I interferon formation.

The three mammalian InsP_3_R isoforms are 60–70% identical in sequence and share a common domain structure [7, 8]. It has been proposed that the C-terminal cytoplasmic region of the InsP_3_R channel functions as the gatekeeper [11–13]. Several proteins, such as Bcl2, Bcl-X_L_, polyglutamine expanded huntingtin (Htt^exp^), huntingtin-associated protein 1A, and protein kinase C substrate 80 K-H (80 K-H), have been shown to interact with this region and regulate the channel activity of InsP_3_R [8]. Here, we found that SEC5 directly binds to the α-helix H1 of InsP_3_R (rat InsP_3_R1: aa 2571–2606), which provides a mechanistic linkage between InsP_3_R-mediated Ca^2+^ levels and antifungal innate immunity. A high-resolution cryo-electron microscopy (cryo-EM) structure of rat type 1 InsP_3_R was recently solved [13], which demonstrates that the sixth transmembrane (TM6) helix, which we have referred to as H1 here, extends into the cytoplasm. In the structure, the phenylalanine at aa 2585 (F2585) appears to form the channel gate. It is not obvious how SEC5 could access this site. Nevertheless, interaction with such a critical site in the channel would be predicted to influence channel gating, as we observed.

Type I interferons (IFNs-I) are classical antiviral cytokines, which can also be induced by intracellular and extracellular bacterial invasions through the activation of the interferon regulatory factors (IRF-3, IRF-5, and IRF-7) [49–51]. It has been recently reported that fungal infections also induce IFNs-I production, and defective IFNs-I responses render both mice and humans susceptible to systemic candidiasis [21–24]. Several PRRs, such as the toll-like receptors (TLR7 and TLR9), Dectin-1, and the interferon regulatory factor family, namely, IRF-1, IRF-3, IRF-5 and IRF-7, have been reported to regulate IFN-β production upon *C. albicans* infection [21–23]. Although TBK1 can directly phosphorylate the IRF-3, IRF-5, and IRF-7 transcription factors, enhancing their transactivating potential, the role of TBK1 in antifungal innate immunity is elusive. Chien et al. found that the RalB-SEC5-TBK1 activation complex supported the host defense response, triggered by either extracellular double-stranded RNA (dsRNA) or exposure to Sendai virus [18]. Similarly, we found that the InsP_3_R-SEC5-TBK1 complex regulated the production of IFN-β in response to *C. albicans* stimulation, which was independent of RalB (Additional file 10: Figure S8). Although we focused on the ability of InsP_3_Rs to release Ca^2+^ from intracellular stores, InsP_3_Rs may also regulate associated proteins independently of their ability to release Ca^2+^. Here, we found that InsP_3_R-H1 and SEC5-rbd compete for a common binding site on SEC5 (here referred to as SEC5-2), suggesting that the direct interaction between InsP_3_Rs and SEC5 released the SEC5-rbd domain, thus forming an “open conformation” for TBK1 docking and facilitating the activation of TBK1. We also found that the enzymatic activity of TBK1 was not Ca^2+^ dependent. In agreement with this, ER stress has been shown to augment transcription of select IRF-3-regulated genes, but increasing cytoplasmic Ca^2+^ alone was insufficient to phosphorylate IRF-3 [52], indicating that the direct interaction of InsP_3_R and SEC5 may be involved in the activation of IRF-3. Interestingly, Bourgeois et al. reported that *Candida*-induced IFN-β release required phagocytosis [22]. In the present study, we have shown that the interaction of SEC5 with InsP_3_R modulates both *C. albicans*-induced phagocytosis and IFN-β production.

## Conclusions

In summary, we have defined the mechanistic basis by which InsP_3_R-mediated Ca^2+^ release regulates innate immunity against *C. albicans* and revealed that the InsP_3_R-SEC5 effector complex is required for the proximal activation of TBK1 in response to *C. albicans* infection*.* The formation of the InsP_3_R-SEC5 complex not only promoted InsP_3_R-mediated [Ca^2+^]_c_ elevation, which is necessary for macrophage phagocytosis, but also enhanced TBK1 activity to activate the IRF-3-dependent type I interferon innate immune response against fungal infection. Our investigation has suggested a novel molecular mechanism to regulate the innate immune response against *C. albicans* infection.

## Methods

### Yeast two-hybrid screening

The yeast two-hybrid experiment was performed by Jun Yang following the protocol as previously described [53] in Dr. J. Kevin Foskett’s laboratory at the University of Pennsylvania, Perelman School of Medicine. Briefly, a cDNA fragment encoding the carboxyl terminus of rat InsP_3_R1 aa 2590-C was cloned into the pLexA vector (Clontech) to be used as a bait to screen a human fetal brain cDNA library (Invitrogen). Library screening was performed following the company’s instructions. From 6 × 10^6^ primary transformants we discovered 14 positive colonies. The library plasmids were recovered using a yeast plasmid extraction kit (Qiagen). The prey sequences were identified by the DNA sequence facility of the University of Pennsylvania. Positive plasmids were confirmed by co-transformation of the library and bait plasmids for comparison with non-specific interactions by co-transformation of the library plasmid and the empty pLexA vector.

### Cell culture

Murine macrophage RAW264.7 and human embryonic kidney (HEK) 293 cells were purchased from ATCC, and grown in Dulbecco’s modified Eagle’s medium (DMEM) and minimum essential medium containing 10% fetal bovine serum (FBS), respectively. Chicken B-lymphoid cell line DT40 cells were derived from Dr. J. Kevin Foskett’s laboratory. They were maintained in RPMI 1640 containing 10% FBS, 1% chicken serum, 1% Antibiotic-Antimycotic at 37°C, 95% humidity, 5% CO_2_.

### Mouse macrophage differentiation and culture

Primary cultures of BMDMs from C57BL/6 mice were prepared as previously described [54]. In brief, bone marrow cells were aseptically collected from the female mice aged 6–8 weeks by flushing the euthanized mice’s leg bones with phosphate-buffered saline (PBS). The cells were then incubated in red cell lysis buffer (155 mM NH_4_Cl, 10 mM NaHCO_3_, and 0.1 mM ethylenediaminetetraacetic acid (EDTA)) before centrifuging. The cells were cultured for 7 days in DMEM containing 20% FBS, 100 μg/ml streptomycin, 55 mM mercaptoethanol, 100 U/ml penicillin, and 30% conditioned media from L929 cells expressing macrophage colony-stimulating factor (M-CSF). Non-adherent cells were removed. Flow cytometry analysis indicated that the harvested cell population contained 86–95% CD11b + F4/80 + cells.

### Plasmid construction

Human full-length SEC5 cDNA and IRF-3 cDNA were purchased from YR Gene. Plasmids were constructed according to standard protocols, i.e., by cloning the cDNA corresponding to the residues of interest into vectors including pET28a, pCDNA3.1-V5-His (Life Tech), pECFP-N1 (Clontech), and pGEX-6P-1 (GE). Restriction enzymes for cloning were purchased from New England Biolabs or Takara.

### *C. albicans* infection model and fungal burden

For in vivo *C. albicans* infection, a group of C57B/L6 female mice aged 6–8 weeks were injected via lateral tail vein with 1 × 10^5^ CFUs of *C. albicans* in 200 μl sterile saline. One hour later, the mice were injected i.p. with H1-Tat peptide or H1-scrambled-TAT peptide at 2 mg/kg, or with SEC5-2 peptide at 50 μg/kg. Kidney CFU assays were performed 2 days after infection. The kidneys were removed, weighed, and homogenized in sterile PBS in a tissue grinder. The number of viable *Candida* cells in the tissues was determined by plating serial dilutions on SD agar plates. The CFUs were counted after 24 h of incubation at 30°C and expressed as CFUs per gram of tissue.

### Recombinant protein purification from *E. coli* and GST pull-down assays

Glutathione *S*-transferase (GST) fusion proteins were expressed in BL21 (DE3)pLysS *E. coli* and purified with Glutathione Sepharose 4B following the manufacturer’s protocol (GE). His-tagged proteins were expressed in BL21 (DE3)pLysS *E. coli* using pET28A vectors and purified using Ni-columns according to the manufacturer’s protocol (Qiagen). Purified proteins were dialyzed against 1 × Dulbecco’s PBS (DPBS) buffer. Unless specified, pull-down assays and western blotting were performed according to standard protocols. Cellular lysates, prepared using 1 × DPBS containing 1% Triton X-100, 0.5–1 mg/ml total cellular protein (dependent on expression), in a total volume of 1 ml, were mixed with 20 μg GST fusion protein bound to beads and incubated at 4°C while rotating for 2 h. The beads were spun down and washed thrice using lysate buffer. Bound proteins were eluted using 1 × loading buffer and detected by western blotting.

### Co-immunoprecipitation

RAW264.7 cells, stimulated with *C. albicans* (multiplicity of infection (MOI) = 10) or not, were lysed using 1 × DPBS containing 1% Triton X-100. Ten micrograms of rabbit InsP_3_R1 or InsP_3_R3 antibodies, or an equivalent amount of rabbit immunoglobulin G (IgG) isotype control (Sigma; #18140), was conjugated with 30 μl of a 50% slurry of protein G agarose resin according to the manufacturer’s instructions (Yeasen; #36405ES08). Then, the antibody-conjugated agarose resin was incubated with protein lysates (500 mg of protein) with gentle agitation for 2 h. The beads were retained after three washes with PBS. Next, 1 × loading buffer was added to dissociate immunoprecipitates, and western blotting was performed accordingly.

### Acceptor photobleaching fluorescence resonance energy transfer (FRET)

Acceptor photobleaching FRET was measured by confocal microscopy (A1R, Nikon, Japan) according to a previously described method [29, 30]. Energy transfer was detected as an increase in donor fluorescence (Alexa Fluor 488) following the complete photobleaching of the Cy3 acceptor molecules. The amount of energy transfer at co-localization areas was calculated as the percentage increase in donor fluorescence, and non-co-localization areas were selected as negative controls. The imaging sequence was as follows: First, donor and acceptor channels were sequentially scanned at low laser intensity before the acceptor was bleached with the 561-nm laser line at 70% intensity, and the donor and acceptor channels were scanned again at low laser intensity. Acquired images were exported to 8-bit TIFF format, and FRET efficiency was analyzed using Nikon NIS-Elements software.

### Immunofluorescence staining

BMDM cells at 7 × 10^5^ were placed on 12-mm coverslips (Fisher Scientific, UK) and incubated with *C. albicans* at 37°C in 5% CO_2_ for 60 min. The cells were fixed using 4% paraformaldehyde (PFA) in DPBS, followed by blocking and permeabilization with 0.1% IGEPAL (Sigma, St. Louis, MO, USA) in DPBS with 2% bovine serum albumin (BSA) (Amresco, Solon, OH, USA). Primary antibodies, diluted in 2% BSA, were applied at 4°C overnight. The cells were subsequently washed four times with DPBS before being incubated with the appropriate secondary antibodies (Invitrogen, Thermo Fisher Scientific, Waltham, MA, USA) diluted in 2% DPBS. The coverslips were washed four times with DPBS before being mounted using Vectashield. Confocal images were acquired with a Nikon A1R confocal system. Pearson’s correlation coefficients were calculated using the NIS-Elements AR 4.5 software to show the overlap between Alexa Fluor 488 and Cy3 at each co-localization area.

### Calcium imaging

HEK293 cells were seeded in 35-mm dishes and incubated with 2 μM Fura-2 AM (Invitrogen, Thermo Fisher Scientific, MA, USA) in Hank’s balanced salt solution (HBSS, Sigma, St. Louis, MO, USA) containing 0.04% Pluronic F-127 (Sigma) for 30 min, in normal culture media, at 37°C and 5% CO_2_. The cells were then washed and continuously perfused with HBSS containing 1.8 mM CaCl_2_ and 0.8 mM MgCl_2_, pH 7.4. In the experiments to measure calcium imaging, the cells were perfused with HBSS containing 50 μM carbachol. Fura-2 was alternately excited at 340 and 380 nm, and the emitted fluorescence, filtered at 510 nm, was recorded using Nikon NIS-Elements software. Individual cells were chosen as different regions of interest (ROIs), and the ratio (340/380) of each ROI in all time frames was obtained after background correction. Dye calibration was performed, and ratios were then converted to cytosolic Ca^2+^ concentrations by the Grynkiewicz equation: [Ca^2+^] = K_d_*β*(R-Rmin)/(Rmax-R) [55].

### Nuclear patch-clamp recording of InsP_3_R

Nuclear patch-clamp recording was performed as previously described [35–37]. Briefly, DT40TKO-InsP3R3 nuclei were isolated by homogenizing cells in nuclei isolation solution. Then, 80 μl of cell homogenate was added to 2 ml of bath solution. Single InsP_3_R channels were detected using a HEKA EPC-10 amplifier (HEKA Elektronik) and pipettes filled with pipette solution. Peptides were directly added to the patch pipette solution. Single-channel analysis was performed using QuB software (University of Buffalo).

### Transfection with siRNA and plasmids

The cells were seeded in 6-well plates (1 × 10^6^ cells/well) in 1 ml DMEM containing 10% FBS before being transfected with 120 pmol siRNA against SEC5, non-targeting control siRNA, or 2 μg SEC5-EGFP or pcDNA-EGFP control plasmids, using Lipofectamine 2000 transfection reagent (Invitrogen) according to the manufacturer’s protocol. The cells were lysed 48 h after transfection, and the relative intensities of SEC5 protein were determined by western blotting.

### Phagocytosis assays

Phagocytosis assays were performed using the standard protocol with modifications [38]. Briefly, *C. albicans* (SC5314) cells were added to 1 × 10^6^ BMDM macrophages in glass-based imaging dishes at an MOI of 5. Video microscopy was performed using a Nikon ECLIPSE Ti microscope in a 37°C chamber, and images were captured at 20-ss intervals over a 1-h period using an EMCCD camera. Live-cell video microscopy enables internalization of *C. albicans* to be visualized without the need to stain external *Candida*. At least three independent experiments were performed for each group and at least three videos were analyzed from each experiment using ImageJ analysis software.

### In vitro TBK1 kinase assays

TBK1 kinase assays were performed using the standard protocol with modifications [56]. Endogenous TBK1 from COS-1 cells was added to each kinase activity assay in a buffer containing 25 mM 4-(2-hydroxyethyl)-1-piperazineethanesulfonic acid (HEPES) (pH 7.6), 0.1 mM sodium vanadate, 20 mM β-glycerophosphate, 10 mM MgCl_2_, 50 mM NaCl, and 100 μM ATP. For each reaction, 20 μg of purified recombinant GST-tagged IRF-3-C was used as a substrate for TBK1, and the reactions were carried out for 30 min at 30°C. The reactions were terminated by adding sample buffer. The phosphorylation of recombinant GST-IRF3-C was visualized by sodium dodecyl sulfate-polyacrylamide gel electrophoresis (SDS-PAGE) with phosphor-IRF-3 (Ser396) antibodies (Cell Signaling Technology, Cat#29047), and the equivalent protein concentrations were confirmed by immunoblotting with anti-TBK1 and anti-GST antibodies.

### Cytokine measurements

The enzyme-linked immunosorbent assay kits for IFN-β were purchased from NeoBioscience. All samples were measured in triplicate according to the manufacturer’s protocol.

### Statistical analysis

At least three biological replicates were performed for all experiments, unless otherwise indicated. Individual data for each experiment are provided as Additional file 11. Student’s *t* test was used for statistical analyses of paired observations. Differences between means were accepted as statistically significant at the 95% level (*p* < 0.05).

### Antibodies

The primary antibodies used for immunoblotting were rabbit monoclonal anti-TBK1 (Cell Signaling Technology, Danvers, MA, USA, Cat# 3504S RRID:AB_225566,1:1000 dilution), rabbit monoclonal anti-phosphorylated TBK1 (phosphor S172) (Cell Signaling Technology Cat# 5483P RRID:AB_10693472,1:1000), rabbit monoclonal anti-β-actin (Cell Signaling Technology Cat# 4970S RRID:AB_2223172, 1:1000), rabbit monoclonal anti-PCNA (Cell Signaling Technology Cat# 13110 RRID:AB_2636979,1:1000), rabbit monoclonal anti-IRF3 (phosphor Ser396) (Cell Signaling Technology Cat# 4302S RRID:AB_1904036,1:1000), rabbit monoclonal anti-phosphorylated IRF-3 (Cell Signaling Technology Cat# 29047 RRID:AB_823547,1:1000), rabbit polyclonal anti-SEC5 (Sigma-Aldrich, Atlas Antibodies Cat# HPA032093 RRID:AB_10611964, 1:1000), mouse monoclonal anti-InsP_3_R-3 (BD Biosciences, Franklin, NJ, USA,BD Biosciences Cat# 610313 RRID:AB_397705,1:1000), rabbit polyclonal anti-GST (ABclonal Technology, Wuhan, China, ABclonal Technology Cat#AE006,1:1000), and mouse monoclonal anti-His tag (ABclonal Technology Cat#AE003,1:1000).

The antibodies used for immunofluorescence were rabbit monoclonal anti-TBK1 (Abcam, Cambridge, UK, Abcam Cat# ab40676 RRID:AB_776632,1:100 dilution), mouse monoclonal anti-InsP_3_R-3 (BD Biosciences Cat# 610313 RRID:AB_397705, 1:100), rabbit polyclonal anti-SEC5 (Atlas Antibodies Cat# HPA032093 RRID:AB_10611964, 1:100), Alexa Fluor 488-conjugated donkey anti-mouse IgG (H + L) highly cross-adsorbed secondary antibody (Life Technologies, Molecular Probes Cat# A-21202 RRID:AB_141607,1:400), and Cy3-conjugate donkey anti-rabbit IgG, species adsorbed, antibody (Millipore, Burlington, MA, USA, Millipore Cat# AP182C RRID:AB_92588,1:400).

Polyclonal antibody anti-Type 1 InsP_3_R was generated by Abgent Biotech. Co. Ltd. (Shanghai, China) against the peptides RIGLLGHPPHMNVNPQQPA, strictly following Akihiko Tanimura’s method [57]. Antigens were prepared by GL Biochem. Ltd. (Shanghai, China). The specificity of the InsP_3_R1 antibody has been previously validated [57, 58]. We also reconfirmed the specificity of the polyclonal antisera made against the peptides from the type 1 InsP_3_ receptors in chicken DT40 cells with all InsP_3_R isoforms genetically deleted (DT40-KO) and engineered to stably express InsP_3_R type 1 (KO-R1) or type 2 (KO-R2), which were provided by Dr. J. Kevin Foskett. The mouse monoclonal antibody against InsP_3_R3 was purchased from BD Biosciences. Its specificity was confirmed by western blot of DT40-KO stably expressing a mutant InsP_3_R3, which agreed with the results of immunocytochemistry and western blotting assay of InsP_3_R3 by other groups [58, 59].

### Reagents

Inositol-1,4,5-trisphosphate (Cat#10008205) and araguspongin B (Cat#10006797) were from Cayman Chemical (Ann Arbor, MI, USA). The other reagents are indicated as follows:

- SiRNA-SEC5 (Shanghai GenePharma Co., Ltd., Shanghai, China)

5’-GGUCGGAAAGACAAGGCAGTT-3’

- SiRNA-Negative Control (Shanghai GenePharma Co., Ltd., Shanghai, China)
- 5’-UUCUCCGAACGUGUC ACG UTT-3’

ShRNA-SEC5 (Shanghai Genechem Co., Ltd., Shanghai, China)

5’-CCGGGCCGAAGAGATAAAGAGATTACTCGAGTAATCTCTTTATCTCTTCGGCTTTTTT-3’

### Animals

C57BL/6 mice were purchased from Shanghai BK Animal Model Inc. Ltd., China.

## Additional files

Additional file 2: **Figure S1.** Results of the yeast two-hybrid screen. (A) The sequence of the bait. (B) Sequence alignment of the two positive clones (49.JUN.32 and 47.JUN.30) and SEC5. (DOCX 5416 kb)

Additional file 3: **Figure S2.** GST-H1 pulls down purified His-tagged SEC5-2 and SEC5-4 fragments. (A) Coomassie blue-stained gel showing the purity of His-tagged SEC5-2 and SEC5-4. (B) Immunoblots of in vitro pull-down assays using GST-InsP_3_R-H1 and purified His-tagged SEC5-2. (C) Immunoblots of in vitro pull-down assays using GST-InsP_3_R-H1 and purified His-tagged SEC5-4. (DOCX 1448 kb)

Additional file 4: **Figure S3.** InsP_3_R inhibitor araguspongin B (*ARB*) inhibits the carbachol-induced Ca^2+^ elevation. (A) Representative Ca^2+^ traces depicting carbachol-induced ER Ca^2+^ release (*black bar*) under the influence of ARB; experiments were performed similarly to those described in Fig. 3a and b with cells pretreated with 2 μM ARB or dimethyl sulfoxide (DMSO) for 2 h. (B) Quantification of Ca^2+^ peak amplitude; data are summarized as the mean ± standard error of the mean (SEM) from three experiments with at least 100 cells (**p < 0.005, ***p < 0.001). (DOCX 239 kb)

Additional file 5: **Figure S4.** Sequence alignment of the InsP_3_R-H1 region from three types of InsP_3_Rs, H1-TAT, and H1-scrambled TAT control peptide. (DOCX 1262 kb)

Additional file 6: **Figure S5.** Confocal images depicting the relocalization of SEC5 and InsP_3_R in RAW264.7 cells. Representative resting (*upper panel*) or activated (*C. albicans*-infected; *lower panel*) RAW264.7 cells were immunostained with anti-SEC5 (*red*) and anti-InsP_3_R3 antibodies (*green*). (DOCX 335 kb)

Additional file 8: **Figure S6.** Confocal images depicting the localization of TBK1 and InsP_3_R in BMDM cells. (A) Confocal images depicting the co-localization of TBK1 (*red*) and InsP_3_R (*green*) in resting BMDMs (Pearson coefficient = 0.75). (B) XYZ images of BMDMs stimulated with *C. albicans* and stained for InsP_3_R (*red*) and TBK1 (*green*). *White arrows* indicate the phagosome. InsP_3_R and TBK1 are *circular bands* around *C. albican* that is being ingested. (DOCX 470 kb)

Additional file 9: **Figure S7.** Both the binding of SEC5 with InsP_3_R-TM6+C and the enzymatic activity of TBK1 are independent of Ca^2+^. (A) Representative western blot depicting GST pull-downs of SEC5 from mouse brain lysates with different concentrations of Ca^2+^. Coomassie blue-stained gel shows the input of GST-tagged InsP_3_R-TM6+C fragments (*lower panel*). (B) Immunoblots depicting the phosphorylation of recombinant IRF-3-C in the presence of Ca^2+^. (DOCX 4817 kb)

Additional file 10: **Figure S8.** *C. albicans-*induced activation of TBK1 was independent of RalB. Representative immunoblots showing the amount of phosphorylated TBK1 (*p*-*TBK1*) in the cytosolic fraction from RAW264.7 cells at different time points of *C. albicans* stimulation (MOI = 10). Cells were pretreated with SEC5-shRNA, RalB-shRNA, or scramble shRNA (*NC*-*shRNA*). (DOCX 680 kb)

Additional file 11: **Table S1.** The original data of Figs. 1c, 3c, e, 4a, c, d, 5b, c, d, e, and 6a. (XLSX 12 kb)

## Abbreviations

InsP_3_R: inositol 1,4,5-trisphosphate receptors
TBK1: tank-binding kinase 1
IRF-3: interferon regulatory factor 3
SEC5: exocyst complex component 2; *C. albicans*: Candida albicans
PRRs: pattern recognition receptors
TLRs: Toll-like receptors
PAMPs: pathogen-associated molecular patterns
PLCγ2: phospholipase Cγ2
14: endoplasmic reticulum
Par100: inositol 1,4,5-trisphosphate
[Ca2+]_c_: cytosolic Ca_2+_ concentrations
BMDMs: bone marrow-derived macrophages
ARB: araguspongin B
FRET: fluorescence resonance energy transfer
SIM: super-resolution structured illumination microscopy
siRNA: small interfering RNA
IFNs-I: type I interferons
M-CSF: macrophage colony-stimulating factor
GST: Glutathione S-transferase

## References

[1] Brown GD (2012). Hidden killers: human fungal infections. Sci Transl Med.

[2] Kullberg BJ, Arendrup MC (2015). Invasive candidiasis. N Engl J Med.

[3] Netea MG, Joosten LAB, van der Meer JWM, Kullberg B-J, van de Veerdonk FL (2015). Immune defence against Candida fungal infections. Nat Rev Immunol.

[4] Xu S, Huo J, Lee K-G, Kurosaki T, Lam K-P (2009). Phospholipase Cγ2 is critical for Dectin-1-mediated Ca^2+^ flux and cytokine production in dendritic cells. J Biol Chem.

[5] Gorjestani S (2011). Phospholipase Cγ2 (PLCγ2) is key component in Dectin-2 signaling pathway, mediating anti-fungal innate immune responses. J Biol Chem.

[6] Nunes P, Demaurex N (2010). The role of calcium signaling in phagocytosis. J Leukoc Biol.

[7] Foskett JK, White C, Cheung K-H, Mak D-OD (2007). Inositol trisphosphate receptor Ca^2+^ release channels. Physiol Rev.

[8] Prole DL, Taylor CW (2016). Inositol 1,4,5-trisphosphate receptors and their protein partners as signalling hubs. J Physiol Lond.

[9] Vaeth M (2015). Ca^2+^ signaling but not store-operated Ca^2+^ entry is required for the function of macrophages and dendritic cells. J Immunol.

[10] Patterson RL, Boehning D, Snyder SH (2004). Inositol 1,4,5-trisphosphate receptors as signal integrators. Annu Rev Biochem.

[11] Uchida K, Miyauchi H, Furuichi T, Michikawa T, Mikoshiba K (2003). Critical regions for activation gating of the inositol 1,4,5-trisphosphate receptor. J Biol Chem.

[12] Taylor CW, da Fonseca PCA, Morris EP (2004). IP(3) receptors: the search for structure. Trends Biochem Sci.

[13] Fan G (2015). Gating machinery of InsP3R channels revealed by electron cryomicroscopy. Nature.

[14] White C (2005). The endoplasmic reticulum gateway to apoptosis by Bcl-X_L_ modulation of the InsP_3_R. Nat Cell Biol.

[15] Chang M-J (2014). Feedback regulation mediated by Bcl-2 and DARPP-32 regulates inositol 1,4,5-trisphosphate receptor phosphorylation and promotes cell survival. Proc Natl Acad Sci U S A.

[16] Tanaka T, Goto K, Iino M. Diverse functions and signal transduction of the exocyst complex in tumor cells. J Cell Physiol. 2016; 10.1002/jcp.25619. 10.1002/jcp.25619 27669116

[17] Stuart LM (2007). A systems biology analysis of the Drosophila phagosome. Nature.

[18] Chien Y (2006). RalB GTPase-mediated activation of the IκB family kinase TBK1 couples innate immune signaling to tumor cell survival. Cell.

[19] Ishikawa H, Ma Z, Barber GN (2009). STING regulates intracellular DNA-mediated, type I interferon-dependent innate immunity. Nature.

[20] Ishikawa H, Barber GN (2011). The STING pathway and regulation of innate immune signaling in response to DNA pathogens. Cell Mol Life Sci.

[21] Biondo C (2011). Recognition of yeast nucleic acids triggers a host-protective type I interferon response. Eur J Immunol.

[22] Bourgeois C (2011). Conventional dendritic cells mount a type I IFN response against Candida spp. requiring novel phagosomal TLR7-mediated IFN-β signaling. J Immunol.

[23] del Fresno C (2013). Interferon-β production via Dectin-1-Syk-IRF5 signaling in dendritic cells is crucial for immunity to C. albicans. Immunity.

[24] Smeekens SP (2013). Functional genomics identifies type I interferon pathway as central for host defense against Candida albicans. Nat Commun.

[25] Groves E, Dart AE, Covarelli V, Caron E (2008). Molecular mechanisms of phagocytic uptake in mammalian cells. Cell Mol Life Sci.

[26] Czibener C (2006). Ca^2+^ and synaptotagmin VII-dependent delivery of lysosomal membrane to nascent phagosomes. J Cell Biol.

[27] Gafni J (1997). Xestospongins: potent membrane permeable blockers of the inositol 1,4,5-trisphosphate receptor. Neuron.

[28] Adebiyi A (2014). RGS2 regulates urotensin II-induced intracellular Ca^2+^ elevation and contraction in glomerular mesangial cells. J Cell Physiol.

[29] Kinoshita A (2003). Demonstration by FRET of BACE interaction with the amyloid precursor protein at the cell surface and in early endosomes. J Cell Sci.

[30] Herman B, Krishnan RV, Centonze VE (2004). Microscopic analysis of fluorescence resonance energy transfer (FRET). Methods Mol Biol.

[31] Eckenrode EF, Yang J, Velmurugan GV, Foskett JK, White C (2010). Apoptosis protection by Mcl-1 and Bcl-2 modulation of inositol 1,4,5-trisphosphate receptor-dependent Ca^2+^ signaling. J Biol Chem.

[32] Yang J, Vais H, Gu W, Foskett JK (2016). Biphasic regulation of InsP_3_ receptor gating by dual Ca^2+^ release channel BH3-like domains mediates Bcl-x_L_ control of cell viability. Proc Natl Acad Sci U S A.

[33] Luo J, Busillo JM, Benovic JL (2008). M_3_ muscarinic acetylcholine receptor-mediated signaling is regulated by distinct mechanisms. Mol Pharmacol.

[34] Tovey SC, Taylor CW (2013). Cyclic AMP directs inositol (1,4,5)-trisphosphate-evoked Ca^2+^ signalling to different intracellular Ca^2+^ stores. J Cell Sci.

[35] Mak D-OD, Vais H, Cheung K-H, Foskett JK (2013). Isolating nuclei from cultured cells for patch-clamp electrophysiology of intracellular Ca^2+^ channels. Cold Spring Harb Protoc.

[36] Mak D-OD, Vais H, Cheung K-H, Foskett JK (2013). Nuclear patch-clamp electrophysiology of Ca^2+^ channels. Cold Spring Harb Protoc.

[37] Mak D-OD, Vais H, Cheung K-H, Foskett JK (2013). Patch-clamp electrophysiology of intracellular Ca^2+^ channels. Cold Spring Harb Protoc.

[38] Lewis LE, Bain JM, Okai B, Gow NAR, Erwig LP. Live-cell video microscopy of fungal pathogen phagocytosis. J Vis Exp. 2013; 10.3791/50196. 10.3791/50196 PMC3582652 23329139

[39] Sharma S (2003). Triggering the interferon antiviral response through an IKK-related pathway. Science.

[40] Fitzgerald KA (2003). IKKepsilon and TBK1 are essential components of the IRF3 signaling pathway. Nat Immunol.

[41] Huynh KK, Kay JG, Stow JL, Grinstein S (2007). Fusion, fission, and secretion during phagocytosis. Physiology.

[42] Melendez AJ, Tay HK (2008). Phagocytosis: a repertoire of receptors and Ca^2+^ as a key second messenger. Biosci Rep.

[43] Shaw PJ, Qu B, Hoth M, Feske S (2013). Molecular regulation of CRAC channels and their role in lymphocyte function. Cell Mol Life Sci.

[44] Shaw PJ, Feske S (2012). Regulation of lymphocyte function by ORAI and STIM proteins in infection and autoimmunity. J Physiol Lond.

[45] Gagnon E (2002). Endoplasmic reticulum-mediated phagocytosis is a mechanism of entry into macrophages. Cell.

[46] Desjardins M (2003). ER-mediated phagocytosis: a new membrane for new functions. Nat Rev Immunol.

[47] Touret N (2005). Quantitative and dynamic assessment of the contribution of the ER to phagosome formation. Cell.

[48] Nunes P (2012). STIM1 juxtaposes ER to phagosomes, generating Ca^2+^ hotspots that boost phagocytosis. Curr Biol.

[49] Trinchieri G (2010). Type I interferon: friend or foe?. J Exp Med.

[50] Decker T, Müller M, Stockinger S (2005). The yin and yang of type I interferon activity in bacterial infection. Nat Rev Immunol.

[51] Bergstrøm B (2015). TLR8 senses Staphylococcus aureus RNA in human primary monocytes and macrophages and induces IFN-β production via a TAK1-IKKβ-IRF5 signaling pathway. J Immunol.

[52] Liu Y-P (2012). Endoplasmic reticulum stress regulates the innate immunity critical transcription factor IRF3. J Immunol.

[53] Yang J, McBride S, Mak DO, Vardi N, Palczewski K, Haeseleer F, Foskett JK (2002). Identification of a family of calcium sensors as protein ligands of inositol trisphosphate receptor Ca^2+^ release channels. Proc Natl Acad Sci U S A.

[54] Jia X-M (2014). CARD9 mediates Dectin-1-induced ERK activation by linking Ras-GRF1 to H-Ras for antifungal immunity. J Exp Med.

[55] Bootman MD, Rietdorf K, Collins T, Walker S, Sanderson M (2013). Converting fluorescence data into Ca^2+^ concentration. Cold Spring Harb Protoc.

[56] Chien Y, White MA (2008). Characterization of RalB-Sec5-TBK1 function in human oncogenesis. Meth Enzymol.

[57] Tanimura A, Tojyo Y, Turner RJ (2000). Evidence that type I, II, and III inositol 1,4,5-trisphosphate receptors can occur as integral plasma membrane proteins. J Biol Chem.

[58] Li C (2007). Apoptosis regulation by Bcl-x_L_ modulation of mammalian inositol 1,4,5-trisphosphate receptor channel isoform gating. Proc Natl Acad Sci U S A.

[59] Vazquez G, Wedel BJ, Bird GSJ, Joseph SK, Putney JW (2002). An inositol 1,4,5-trisphosphate receptor-dependent cation entry pathway in DT40 B lymphocytes. EMBO J.

