# Correction to: InsP_3_R-SEC5 interaction on phagosomes modulates innate immunity to *Candida albicans* by promoting cytosolic Ca ^2+^ elevation and TBK1 activity

**DOI:** 10.1186/s12915-020-00900-6

**Published:** 2020-11-02

**Authors:** Long Yang, Wenwen Gu, King-Ho Cheung, Lan Yan, Benjamin Chun-Kit Tong, Yuanying Jiang, Jun Yang

**Affiliations:** 1 School of Pharmacy, Second Military Medical University, 325 Guohe Road, Shanghai, 200433 China; 2 NHFPC Key Laboratory of Reproduction Regulation, Shanghai Institute of Planned Parenthood Research, 2140 Xie Tu Road, Shanghai, 200032 China; 3 School of Chinese Medicine, Hong Kong Baptist University, Hong Kong SAR, China; 4 Jinan Military General Hospital, 25 Shifan Road, Jinan, 250031 China; 5 School of Medicine, Tongji University, Shanghai, 200433 China

**Correction to: BMC Biol 16, 46 (2018)**

**https://doi.org/10.1186/s12915-018-0507-6**

Following publication of the original article [1], the authors noticed that Fig. 2 contained an error, accidentally introduced in its preparation. In panel e, the image of the top left-hand blot is incorrect, showing a duplication of the top right-hand blot for His-SEC5–2, instead of an image of the genuine blot for His-SEC5–1. The correct figure is shown below.

**Fig. 2:**
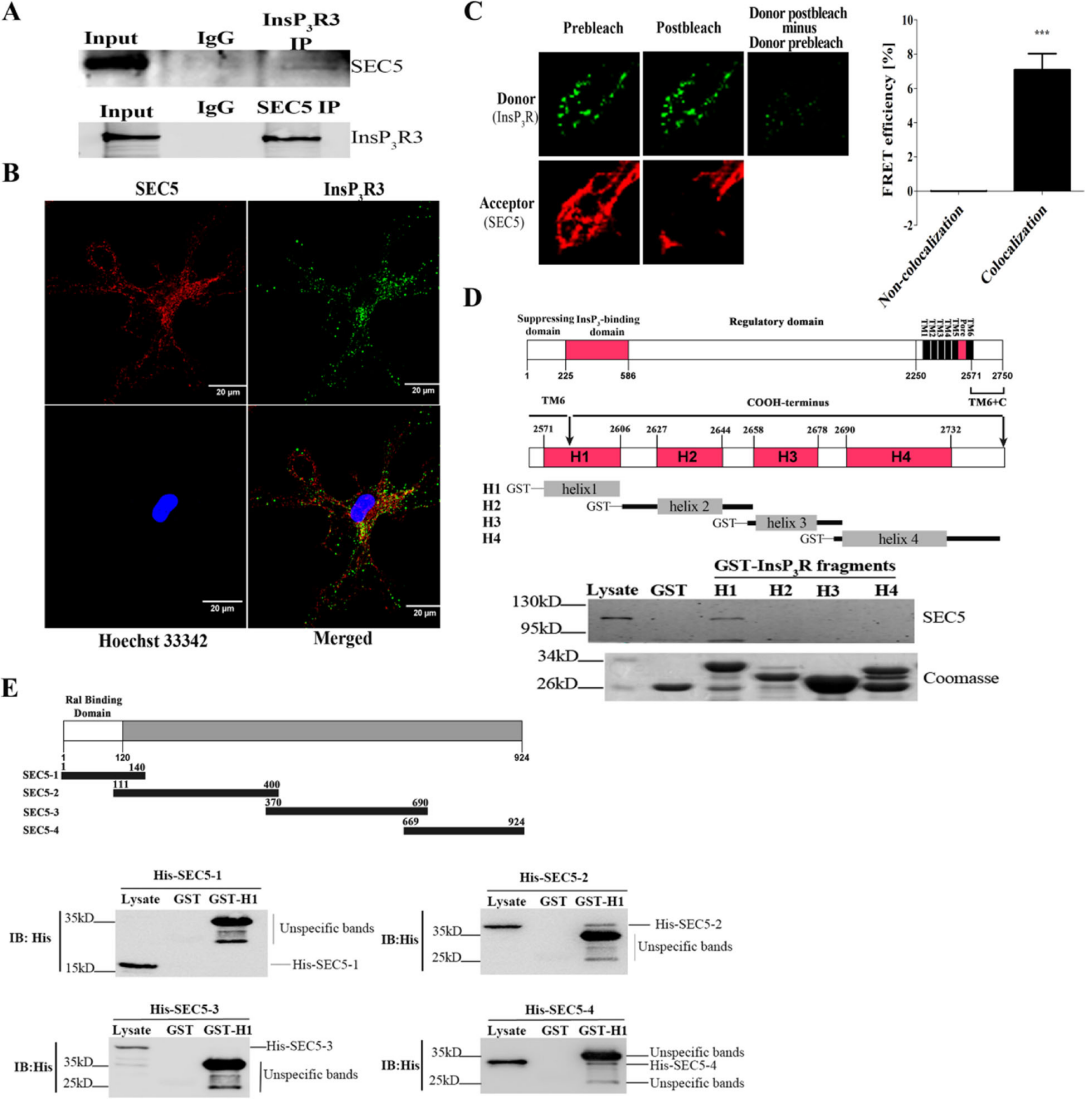
SEC5 interacts with InsP_3_R. **a** Interaction between SEC5 and InsP_3_R3 in RAW264.7 cell lysates. The cell lysates were immunoprecipitated (IP) with control rabbit IgG, anti-SEC5, or anti-InsP_3_R3 antibody. The lysate (Input) and IP samples were analyzed by western blotting with the indicated antibodies. **b** Confocal images showing SEC5 and InsP_3_R co-localization in BMDM cells (Pearson coefficient = 0.71). A representative resting BMDM cell was immunostained with anti-SEC5 (red) and anti-InsP_3_R3 antibodies (green). **c** Representative images of FRET donor (Alexa Fluor 488) and acceptor (Cy3). FRET pair intensities before and after Cy3 was photobleached are shown (left and middle columns). The histogram summarizes FRET efficiency in multiple regions of interest (data are summarized as the mean ± SEM; *n* = 16 for each pair of samples; ***p < 0.001). **d** Schematic illustration of the functional domains of rat InsP_3_R type 1 and GST fusion proteins (H1 to H4) of the InsP_3_R C-terminus (aa 2573–2749) used in SEC5 pull-down assays. A representative western blot depicting the GST pull-downs of SEC5 from RAW267.4 cell extracts is shown. The Coomassie blue-stained gel shows input GST-tagged H1 to H4 fragments. **e** Schematic diagram of recombinant SEC5 fragments used for testing interactions with InsP_3_R H1 helices (upper panel). Representative in vitro pull-down assays depict the interaction of different His-tagged SEC5 fragments with GST-InsP_3_R-H1

The text discussing these data in the original article was based on the genuine data and does not need correction.
