# Supplementary figures and images for: InsP3R-SEC5 interaction on phagosomes modulates innate immunity to Candida albicans by promoting cytosolic Ca2+ elevation and TBK1 activity
Source: BMC Biol. 2018 Apr 27;16:46. doi: 10.1186/s12915-018-0507-6 (PMC5921305; doi:10.1186/s12915-018-0507-6)

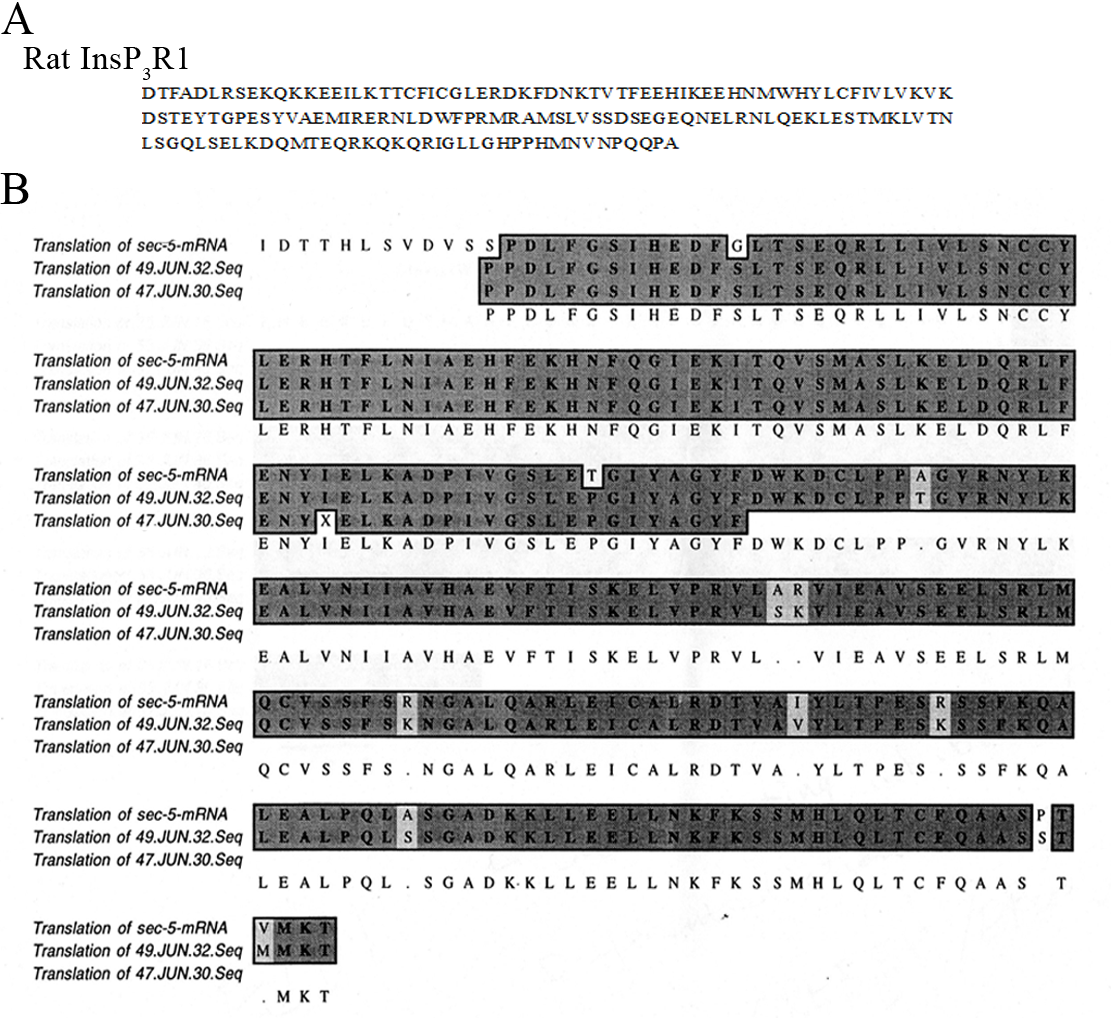

Supplement: Supplementary file 3 — Figure S1. Results of the yeast two-hybrid screen. (A) The sequence of the bait. (B) Sequence alignment of the two positive clones (49.JUN.32 and 47.JUN.30) and SEC5. (DOCX 5416 kb) [file 12915_2018_507_MOESM2_ESM.docx]

**
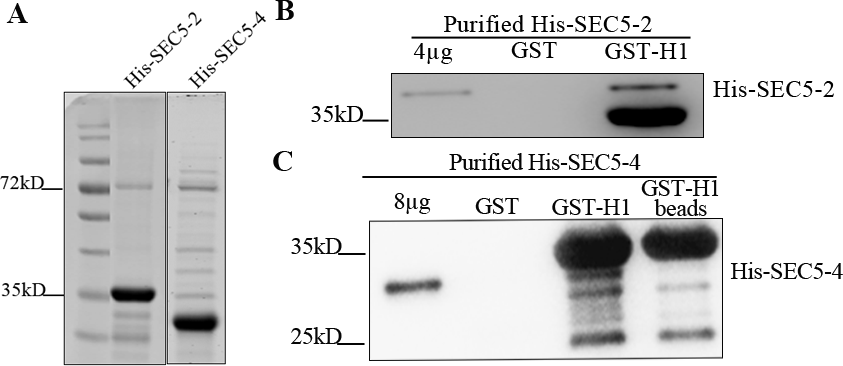
**

Supplement: Supplementary file 4 — Figure S2. GST-H1 pulls down purified His-tagged SEC5-2 and SEC5-4 fragments. (A) Coomassie blue-stained gel showing the purity of His-tagged SEC5-2 and SEC5-4. (B) Immunoblots of in vitro pull-down assays using GST-InsP3R-H1 and purified His-tagged SEC5-2. (C) Immunoblots of in vitro pull-down assays using GST-InsP3R-H1 and purified His-tagged SEC5-4. (DOCX 1448 kb) [file 12915_2018_507_MOESM3_ESM.docx]

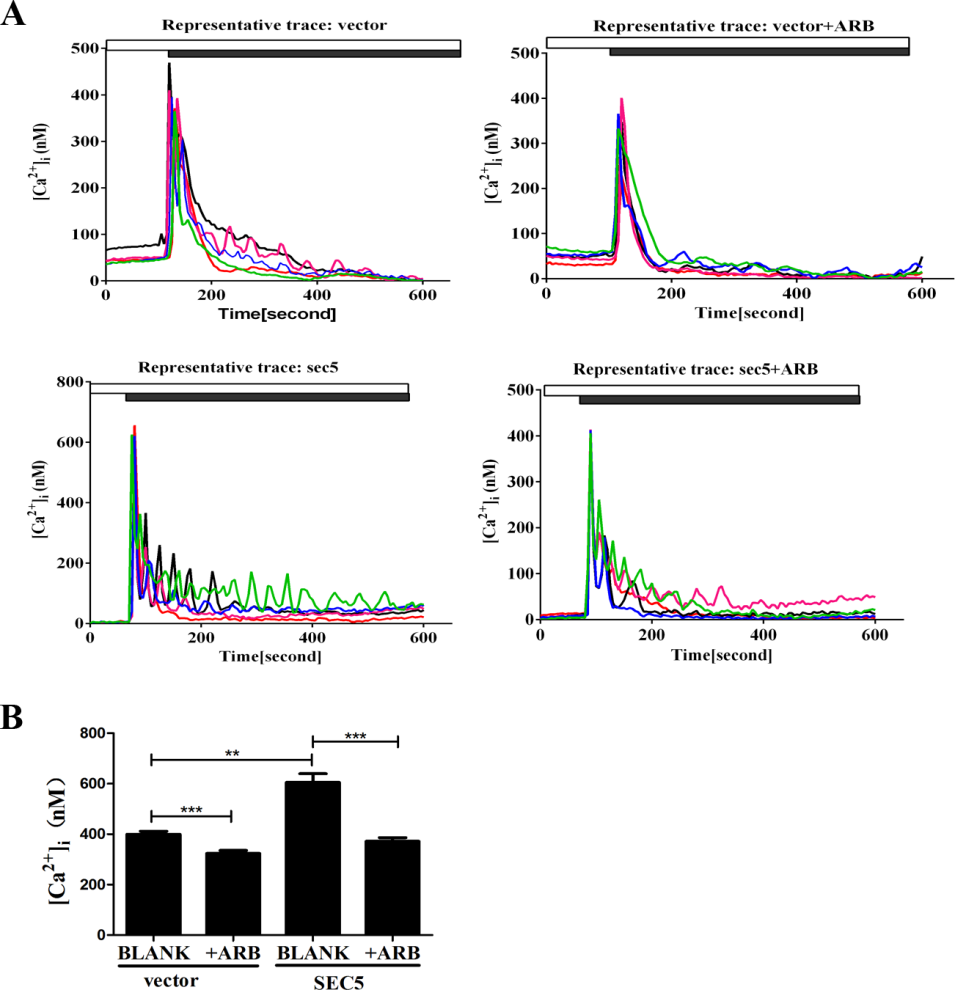

Supplement: Supplementary file 5 — Figure S3. InsP3R inhibitor araguspongin B (ARB) inhibits the carbachol-induced Ca2+ elevation. (A) Representative Ca2+ traces depicting carbachol-induced ER Ca2+ release (black bar) under the influence of ARB; experiments were performed similarly to those described in Fig. 3a and b with cells pretreated with 2 μM ARB or dimethyl sulfoxide (DMSO) for 2 h. (B) Quantification of Ca2+ peak amplitude; data are summarized as the mean ± standard error of the mean (SEM) from three experiments with at least 100 cells (**p < 0.005, ***p < 0.001). (DOCX 239 kb) [file 12915_2018_507_MOESM4_ESM.docx]

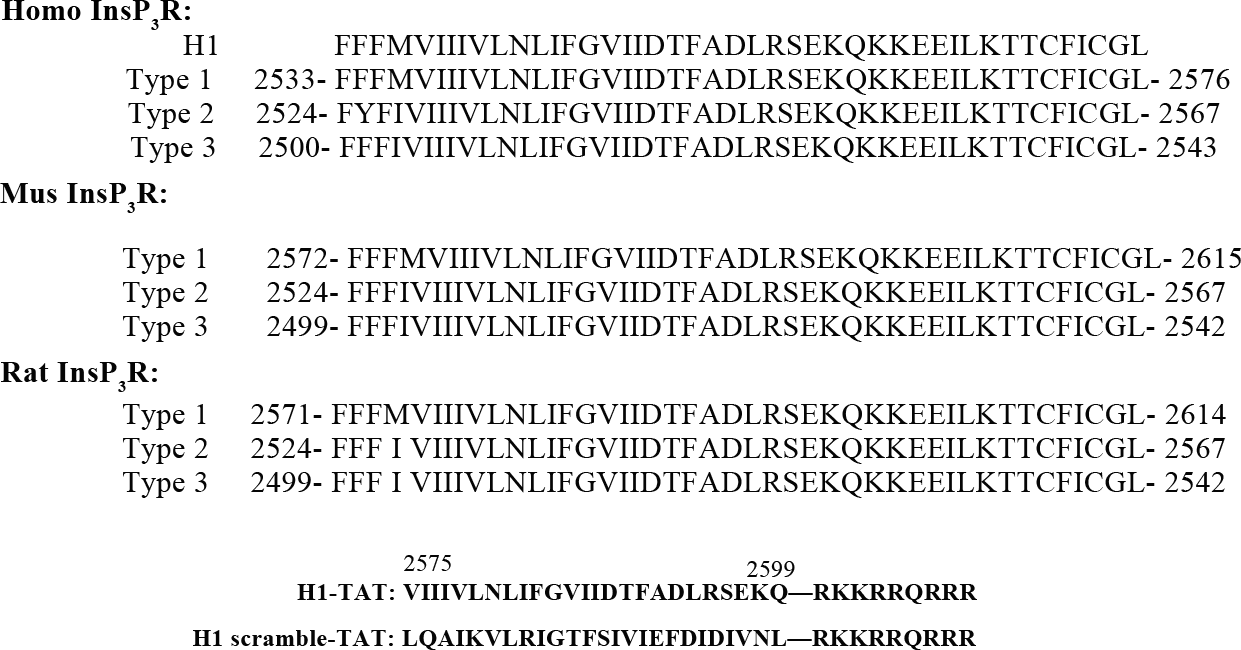

Supplement: Supplementary file 6 — Figure S4. Sequence alignment of the InsP3R-H1 region from three types of InsP3Rs, H1-TAT, and H1-scrambled TAT control peptide. (DOCX 1262 kb) [file 12915_2018_507_MOESM5_ESM.docx]

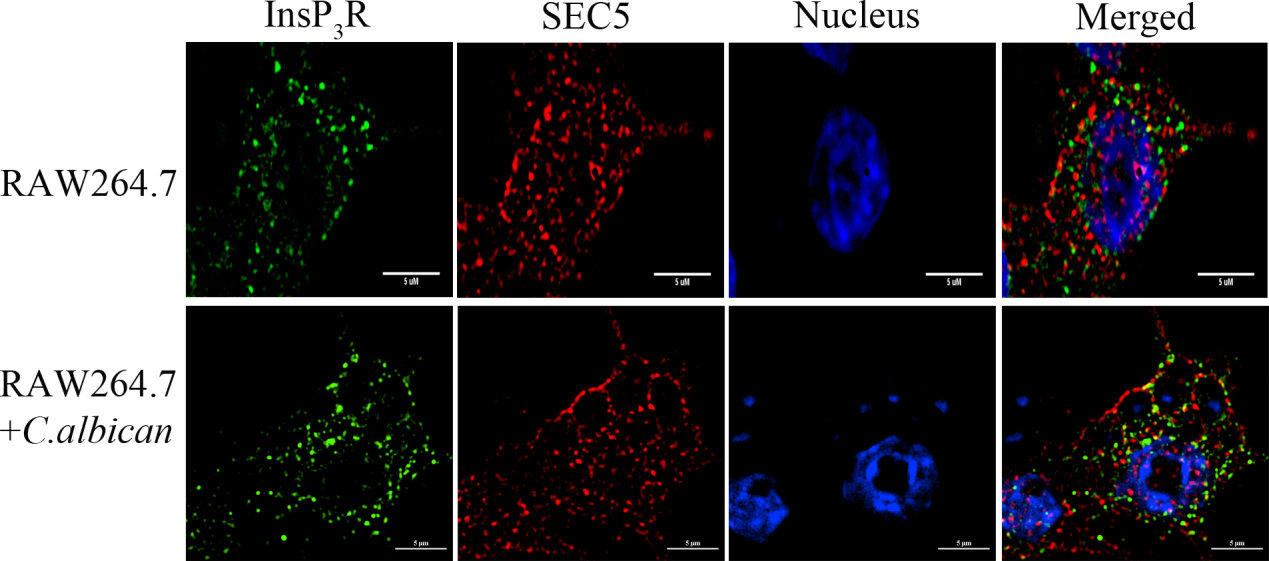

Supplement: Supplementary file 7 — Figure S5. Confocal images depicting the relocalization of SEC5 and InsP3R in RAW264.7 cells. Representative resting (upper panel) or activated (C. albicans-infected; lower panel) RAW264.7 cells were immunostained with anti-SEC5 (red) and anti-InsP3R3 antibodies (green). (DOCX 335 kb) [file 12915_2018_507_MOESM6_ESM.docx]

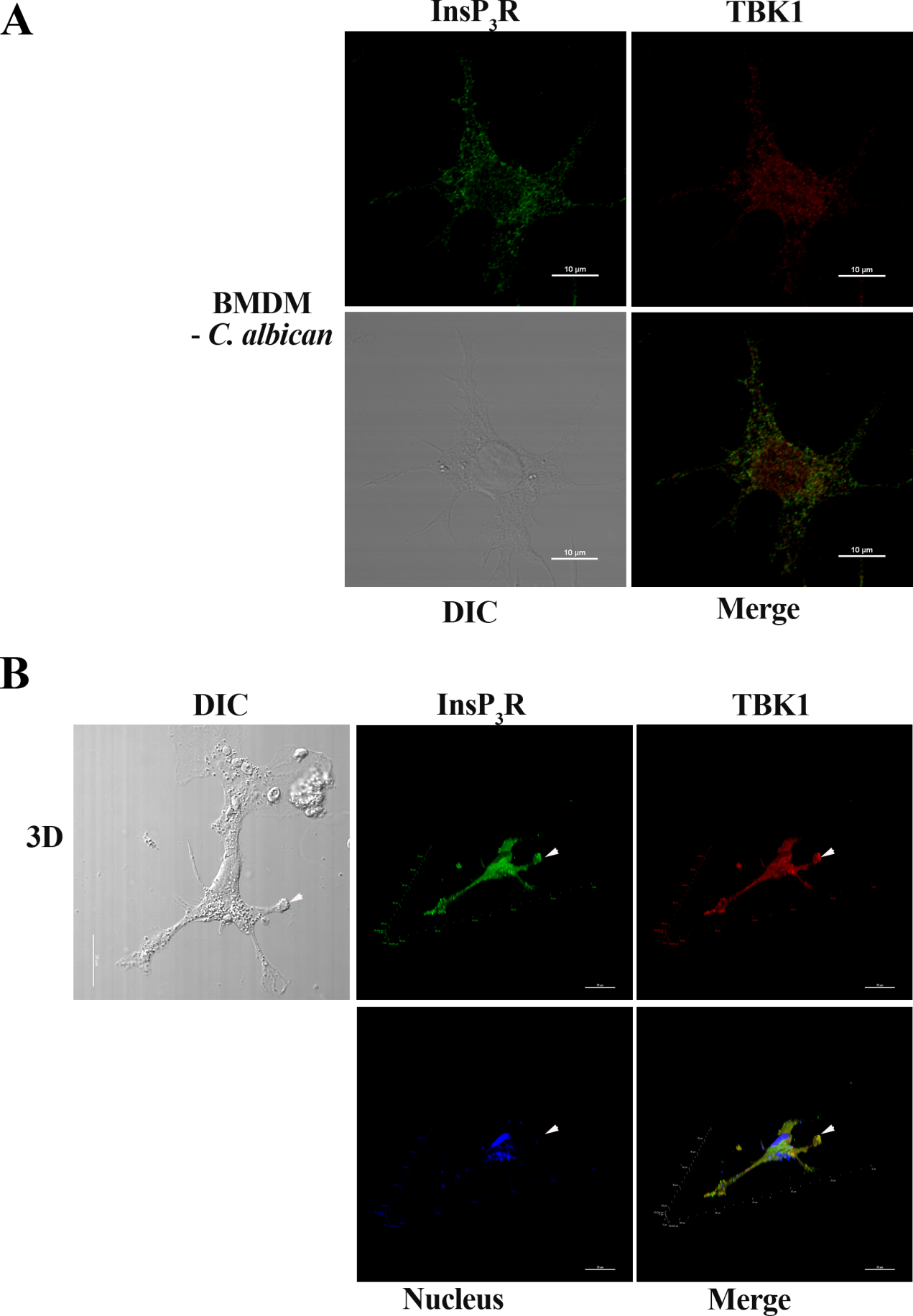

Supplement: Supplementary file 8 — Figure S6. Confocal images depicting the localization of TBK1 and InsP3R in BMDM cells. (A) Confocal images depicting the co-localization of TBK1 (red) and InsP3R (green) in resting BMDMs (Pearson coefficient = 0.75). (B) XYZ images of BMDMs stimulated with C. albicans and stained for InsP3R (red) and TBK1 (green). White arrows indicate the phagosome. InsP3R and TBK1 are circular bands around C. albican that is being ingested. (DOCX 470 kb) [file 12915_2018_507_MOESM8_ESM.docx]

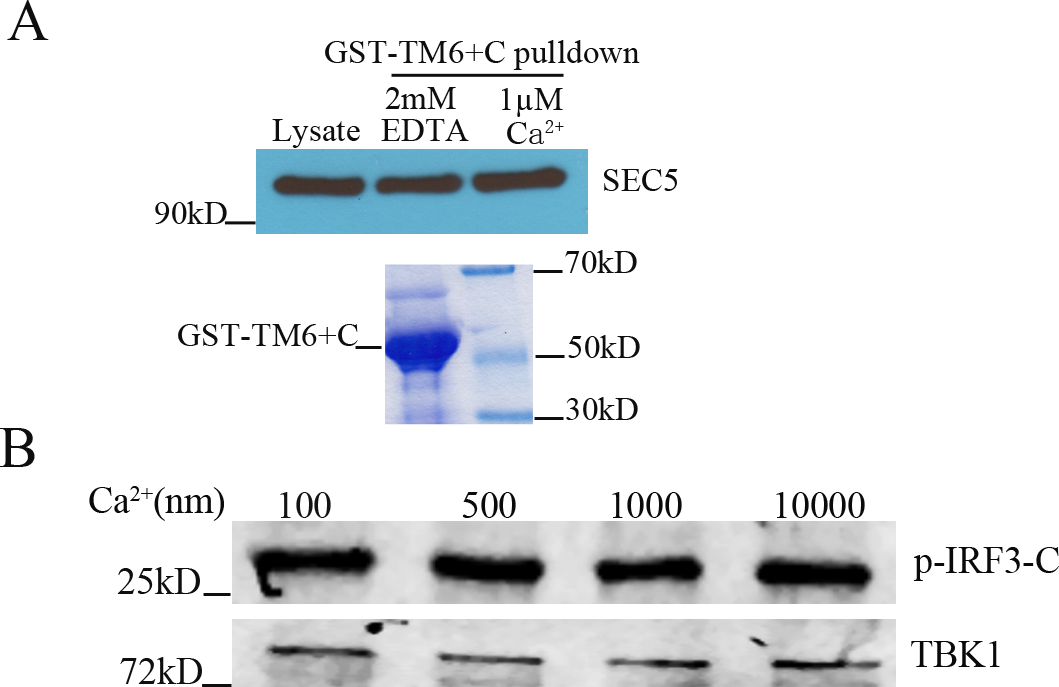

Supplement: Supplementary file 9 — Figure S7. Both the binding of SEC5 with InsP3R-TM6+C and the enzymatic activity of TBK1 are independent of Ca2+. (A) Representative western blot depicting GST pull-downs of SEC5 from mouse brain lysates with different concentrations of Ca2+. Coomassie blue-stained gel shows the input of GST-tagged InsP3R-TM6+C fragments (lower panel). (B) Immunoblots depicting the phosphorylation of recombinant IRF-3-C in the presence of Ca2+. (DOCX 4817 kb) [file 12915_2018_507_MOESM9_ESM.docx]

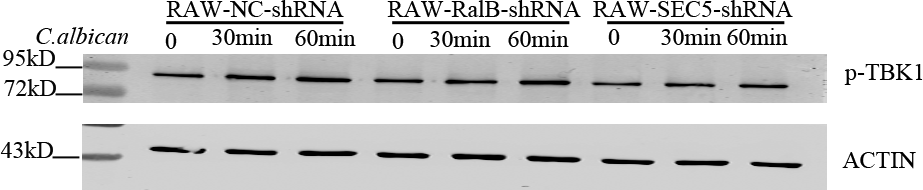

Supplement: Supplementary file 10 — Figure S8. C. albicans-induced activation of TBK1 was independent of RalB. Representative immunoblots showing the amount of phosphorylated TBK1 (p-TBK1) in the cytosolic fraction from RAW264.7 cells at different time points of C. albicans stimulation (MOI = 10). Cells were pretreated with SEC5-shRNA, RalB-shRNA, or scramble shRNA (NC-shRNA). (DOCX 680 kb) [file 12915_2018_507_MOESM10_ESM.docx]
